# In Situ Generation of Ultrathin MoS_2_ Nanosheets in Carbon Matrix for High Energy Density Photo‐Responsive Supercapacitors

**DOI:** 10.1002/advs.202201685

**Published:** 2022-07-07

**Authors:** Zhenbin Tang, Juguo Dai, Wenkang Wei, Zhi Gao, Zhixuan Liang, Chenzhi Wu, Birong Zeng, Yiting Xu, Guorong Chen, Weiang Luo, Conghui Yuan, Lizong Dai

**Affiliations:** ^1^ College of Materials Xiamen University Xiamen 361005 P. R. China; ^2^ Fujian Provincial Key Laboratory of Fire Retardant Materials Xiamen University Xiamen 361005 P. R. China

**Keywords:** 2D semiconductor, boronate ester polymer, carbon materials, photo‐response, supercapacitors

## Abstract

Stimuli‐responsive supercapacitors have attracted broad interest in constructing self‐powered smart devices. However, due to the demand for high cyclic stability, supercapacitors usually utilize stable or inert electrode materials, which are difficult to exhibit dynamic or stimuli‐responsive behavior. Herein, this issue is addressed by designing a MoS_2_@carbon core‐shell structure with ultrathin MoS_2_ nanosheets incorporated in the carbon matrix. In the three‐electrode system, MoS_2_@carbon delivers a specific capacitance of 1302 F g^−1^ at a current density of 1.0 A g^−1^ and shows a 90% capacitance retention after 10 000 charging‐discharging cycles. The MoS_2_@carbon‐based asymmetric supercapacitor displays an energy density of 75.1 Wh kg^−1^ at the power density of 900 W kg^−1^. Because the photo‐generated electrons can efficiently migrate from MoS_2_ nanosheets to the carbon matrix, the assembled photo‐responsive supercapacitor can answer the stimulation of ultraviolet‐visible‐near infrared illumination by increasing the capacitance. Particularly, under the stimulation of UV light (365 nm, 0.08 W cm^−2^), the device exhibits a ≈4.50% (≈13.9 F g^−1^) increase in capacitance after each charging‐discharging cycle. The study provides a guideline for designing multi‐functional supercapacitors that serve as both the energy supplier and the photo‐detector.

## Graphical abstract

An in situ approach based on the coordination between boronate ester polymers and metal ions has been developed to generate ultrathin MoS_2_ nanosheets in the carbon matrix, affording MoS_2_@carbon core‐shell particles. Due to the synergistic effect between the semiconductor MoS_2_ and the multi‐element co‐doped carbon matrix, MoS_2_@carbon exhibits outstanding capacitive performance and light‐triggered reversible capacitance evolution.

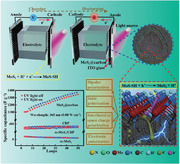

## 1 Introduction

With the rapid development of electrochemically active materials and their processing technology, supercapacitors are recently endowed with more and more functions.^[^ ^1^ ^]^ Indeed, supercapacitors are beyond the scope of energy conversion and storage devices. Recent advances in this area lead to the emergence of stimuli‐responsive supercapacitors (SRSCs),^[^ ^2^ ^]^ which are promising candidates for constructing highly integrated and self‐powered machines. SRSCs can answer to the environmental stimuli through variations in charging mode,^[^ ^3^ ^]^ capacitance,^[^ ^4^ ^]^ shape,^[^ ^5^ ^]^ and color.^[^ ^6^ ^]^ However, charging the supercapacitors by using different stimuli like mechanical force,^[^ ^7^ ^]^ light,^[^ ^8^ ^]^ heat,^[^ ^9^ ^]^ and etc., is just concerning the energy conversion process, but not a kind of classical stimuli‐responsive behavior. Besides, responsive manners like shape deformation and color change can be easily achieved by various materials.^[^ ^10^ ^]^ Therefore, only the stimuli‐triggered capacitance evolution is unique for supercapacitors, but this type of SRSC has not been well explored.

A typical strategy to realize stimuli‐triggered capacitance evolution involves the design of flexible supercapacitors.^[^ ^11^ ^]^ The stimuli‐responsive behavior is mainly attributed to deformable electrolytes and rationally‐designed device structures.^[^ ^12^ ^]^ Some conjugated polymers,^[^ ^13^ ^]^ inorganic semiconductors,^[^ ^14^ ^]^ and metal/conductive polymer composites^[^ ^15^ ^]^ exhibit light‐triggered capacitance evolution, but there are also problems such as low capacitance, slow response, and limited sensitivity. Besides, light‐triggered capacitance evolution has been achieved by utilizing the photo‐thermal effect of graphene and carbon nanotubes.^[^ ^16^ ^]^ Thus, SRSCs based on graphene or carbon nanotubes are mainly sensitive to visible and near‐infrared light. It remains a daunting challenge to fabricate an SRSC that can effectively and reversibly change its capacitance, as well as maintain a high capacitance and stable electrochemical performances.

Theoretically, a combination of photo‐active semiconductors and capacitive materials may be conducive to constructing SRSCs with a photo‐responsive capability. The challenge of this design is that the photo‐generated carriers must efficiently participate in the charging‐discharging process of the electrodes. We envisaged that incorporating a photo‐active semiconductor in the carbon matrix could facilitate the separation and transfer of photo‐generated carriers, and hence enable the effect of carriers on the capacitive performance. As a proof of concept, we designed a MoS_2_@carbon core‐shell structure with photo‐active ultrathin MoS_2_ nanosheets covalently incorporated in the carbon matrix. MoS_2_@carbon was prepared through a pyrolysis‐assisted in situ growth method using MoS_4_^2−^ loaded boronate ester polymer (BP) assemblies as the precursor. Catechol groups in the polymer networks of BP can coordinate with numerous metal ions,^[^ ^17^ ^]^ which promotes the enrichment of MoS_4_^2−^ in BP. After carbonization, MoS_2_ nanosheets were generated in the carbon particles, and C‐Mo bonds were formed between MoS_2_ and the carbon matrix. SRSCs assembled with MoS_2_@carbon displayed not only high specific capacitance, excellent stability, and high energy density and power density, but also showed photo‐responsiveness through the reversible variation of capacitance.

## 2 Results and Discussion

### 2.1 Morphology and Structure of MoS_2_@Carbon

**Figure 1:**
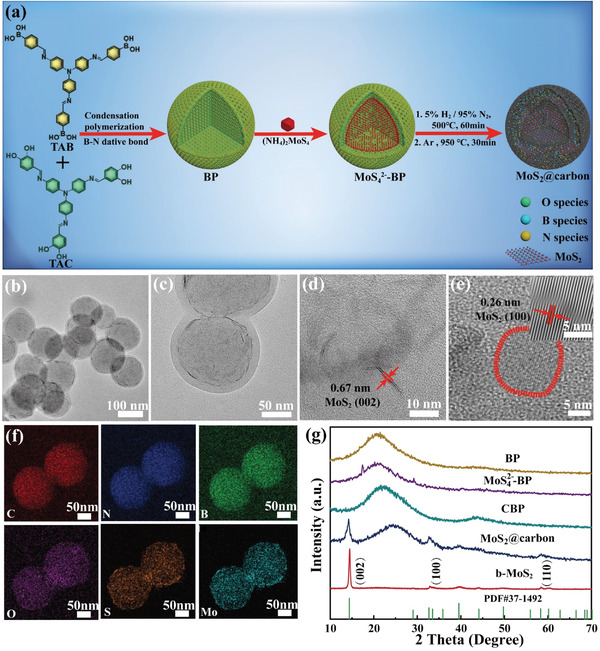
a) Schematic illustration for the synthesis and structure of MoS_2_@carbon. b–e) TEM images of MoS_2_@carbon with different magnifications. f) EDX elemental mappings of MoS_2_@carbon. g) Comparative XRD patterns of BP, MoS_4_^2‐^‐BP, CBP, MoS_2_@carbon, and b‐MoS_2_.

The synthetic procedure of MoS_2_@carbon is illustrated in **Figure** **1**a. A catechol monomer (denoted as TAC) and a boronic monomer (denoted as TAB) were adopted to prepare monodispersed spherical BP (diameter: 199 ± 8 nm, Figures S1a and S2a, Supporting Information). The reaction between TAC and TAB accords to the nucleation polymerization mechanism driven by the formation of B‐N dative bond.^[^ ^17^ ^]^ (NH_4_)_2_MoS_4_ was incorporated in BP through a coordination reaction between MoS_4_^2−^ and catechol groups (Figure S3, Supporting Information).^[^ ^18^ ^]^ As shown in the transmission electron microscope (TEM) images (Figure S1b,c, Supporting Information), MoS_4_^2−^ was concentrated in BP, resulting in a core‐shell structure (denoted as MoS_4_^2‐^‐BP). This is due to the nature of nucleation polymerization, which leads to the formation of polymer chains with low molecular weight and the exposure of more catechol groups (coordination anchors) in BP. MoS_2_@carbon (diameter: 162 ± 6 nm, Figure S2b, Supporting Information) was obtained after the pyrolysis of MoS_4_^2‐^‐BP (Figure 1b). To make the comparison, we also synthesized carbon particles (denoted as CBP, diameter: 157 ± 5 nm, Figures S1d and S2c, Supporting Information) and bulk MoS_2_ (denoted as b‐MoS_2_, Figure S1e, Supporting Information) through the thermolysis of BP and (NH_4_)_2_MoS_4_, respectively. The magnified TEM image (Figure 1c) and elemental mapping pictures (Figure 1f) indicate that ultrathin MoS_2_ nanosheets (about 15 nm, 2 layers of MoS_2_) are dispersed in the carbon matrix to form a core, which is surrounded by a blank sideband (about 13 nm) without MoS_2_ nanosheets. High‐resolution TEM images (Figure 1d,e) illustrate interplanar spacings of 0.67 and 0.26 nm, corresponding to the (002) and (100) crystal planes of MoS_2_, respectively.^[^ ^19^ ^]^ The crystal structure of MoS_2_ in the carbon matrix was verified by the X‐ray diffraction (XRD) pattern (Figure 1g), the diffraction peaks at 14.23°, 32.70°, and 58.54°, are attributed to the (002), (100), and (110) lattice planes of MoS_2_, respectively.

**Figure 2:**
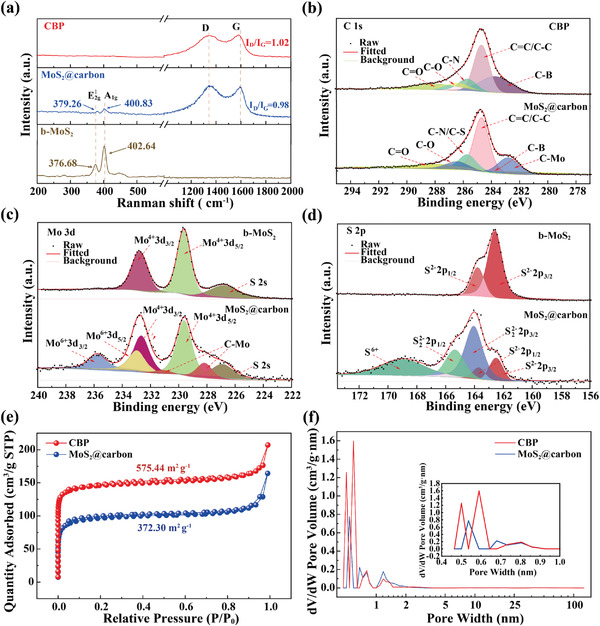
a) Raman spectra of MoS_2_@carbon, CBP, and b‐MoS_2_. b) C 1s XPS spectra of MoS_2_@carbon and CBP. c) Mo 3d and d) S 2p XPS spectra of b‐MoS_2_ and MoS_2_@carbon. e) N_2_ adsorption–desorption isotherm curves and f) pore size distribution curves of MoS_2_@carbon and CBP.

In the Raman spectra (**Figure** **2**a), both MoS_2_@carbon and CBP show characteristic signals of the D band (defects of hexagonal sp^2^ carbon at 1333 cm^−1^) and the G band (sp^2^‐hybridized carbon at 1574 cm^−1^).^[^ ^20^ ^]^ Two peaks at 379.26 and 400.83 cm^−1^, corresponding to the in‐plane ($E_{2g}^{1}$) and vertical plane (A_1g_) of Mo‐S bonds, respectively, indicate the existence of 2H‐MoS_2_ in MoS_2_@carbon. The frequency spacing is about 21.57 cm^−1^, implying that MoS_2_@carbon comprises few‐layered MoS_2_ nanosheets.^[^ ^21^ ^]^ The survey X‐ray photoelectron spectrum (XPS) of MoS_2_@carbon (Figure S4a, Supporting Information) indicates the coexistence of C, N, B, O, Mo, and S elements. The C 1s spectrum (Figure 2b) of MoS_2_@carbon can be deconvoluted into signals with binding energies of 288.0, 286.4, 285.7, 284.7, 283.7, and 282.8 eV, which are attributed to C=O, C‐O, C‐N/C‐S, C=C/C‐C, C‐B, and C‐Mo, respectively.^[^ ^22^ ^]^ However, the C‐Mo signal cannot be identified in the C 1s spectrum of CBP. In the Mo 3d spectrum of MoS_2_@carbon (Figure 2c), the curve‐fitted result displays peaks of Mo^4+^ 3d_3/2_ (231.51 eV), Mo^4+^ 3d_5/2_ (228.65 eV), Mo^6+^ 3d_3/2_ (235.45 eV), Mo^6+^ 3d_5/2_ (232.54 eV), and C‐Mo (228.3, 231.1 eV). Also, a signal of S 2s at 227.0 eV can be observed in Figure 2c.^[^ ^22^ ^]^ The detection of Mo^6+^ signals indicates that a portion of (NH_4_)_2_MoS_4_ is transformed into MoS_3_. The high‐resolution S 2p spectrum of MoS_2_@carbon shows peaks of S^2−^ 2p_1/2_ at 162.3 eV, S^2−^ 2p_3/2_ at 161.2 eV, S_2_^2−^ 2p_1/2_ at 163.8 eV, S_2_^2−^ 2p_3/2_ at 162.6 eV, and S^6+^ at 168.9 eV, revealing the formation of polysulfide after pyrolysis (Figure 2d).^[^ ^23^ ^]^ Additional data involving the O 1s, N 1s, and B 1s spectra of the samples are given in Figure S4b–d, Supporting Information. Doping of oxygen in the carbon matrix can improve surface hydrophilicity and provide active sites to interact with charges.^[^ ^24^ ^]^ For nitrogen doping, it has been verified that the conjugation between the lone‐pair electron of the nitrogen atom and the graphitic *π*‐bond distorts the carbon structure to create defects and available active sites.^[^ ^25^ ^]^ Boron acts as an electron acceptor in the carbon matrix because of its three valence electrons, causing the shift of the Fermi level to the conduction band, as well as modulating the electronic structure of carbon materials.^[^ ^26^ ^]^ The presence of N and O elements improves the wettability of carbon materials in electrolyte solutions.^[^ ^27^ ^]^ We verified this by testing the contact angle of water on the surface of both MoS_2_@carbon and CBP (Figure S5, Supporting Information). Upon contacting the surface of MoS_2_@carbon and CBP, the water drops are completely absorbed within 2 s, indicating their high hydrophilicity. The pore structure of the samples was analyzed using N_2_ adsorption–desorption measurement. Both MoS_2_@carbon and CBP show a typical type I N_2_ adsorption‐desorption isotherm (Figure 2e), and reveal BET surface areas of 372.30 and 575.44 m^2^ g^−1^, respectively. From the pore size distribution diagram (Figure 2f), these two samples are mainly composed of micropores. The t‐Polt micropore volumes of MoS_2_@carbon and CBP are 0.1177 and 0.1828 cm^3^ g^−1^, respectively, with corresponding t‐Polt micropore areas of 301.17 and 473.75 m^2^ g^−1^.

### 2.2 Capacitive Performance of MoS_2_@Carbon

**Figure 3:**
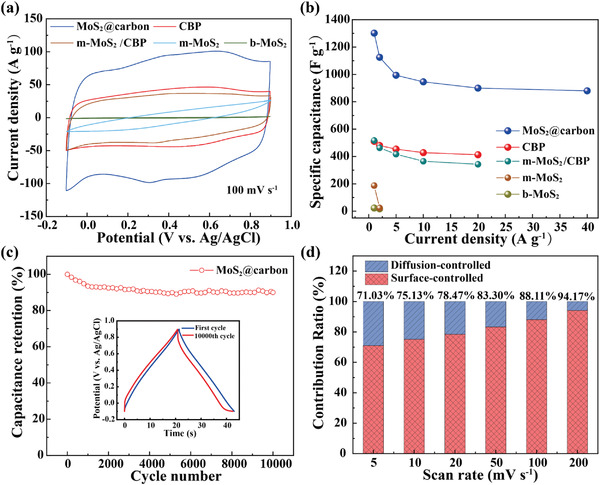
The electrochemical performances of the samples in the three‐electrode system (1 M H_2_SO_4_ electrolyte). a) CV curves and b) specific capacitances of MoS_2_@carbon, CBP, m‐MoS_2_/CBP, m‐MoS_2_, and b‐MoS_2_. c) Cycling performance of MoS_2_@carbon at a current density of 40 A g^−1^, the inset is the GCD plots before and after 10 000 charging‐discharging cycles. d) Diagram of capacitance contributions to the total capacitance at different scanning rates.

MoS_2_@carbon exhibits outstanding capacitive performance in both the three‐electrode system and the two‐electrode system (symmetric and asymmetric type). As the control experiments, we also tested the capacitive performance of CBP, monolayered MoS_2_ (denoted as m‐MoS_2_), and b‐MoS_2_. In particular, the mixture of m‐MoS_2_ and CBP (denoted as m‐MoS_2_/CBP) was fabricated as the control sample. The Mo content of m‐MoS_2_/CBP was 3.04%, which equaled that of MoS_2_@carbon, as measured by inductively coupled plasma‐atomic emission spectrometry (ICP‐AES). In the typical three‐electrode system (mass loading:1.4 mg cm^−2^, electrolyte: 1 M H_2_SO_4_), the cyclic voltammetry (CV) curves (**Figure** **3**a, Figure S6a, Supporting Information) of MoS_2_@carbon exhibit weak redox peaks, and show a dramatically larger integral area than those of CBP, m‐MoS_2_/CBP, m‐MoS_2_, and b‐MoS_2_, when using the same scanning rate. Calculations (details can be found in the Supporting Information) based on the galvanostatic charge–discharge (GCD) curves (Figure S6b,c, Supporting Information) of MoS_2_@carbon give specific capacitances of 1302, 1125, 994, 947, 900, and 881 F g^−1^ at current densities of 1.0, 2.0, 5.0, 10.0, 20.0, and 40.0 A g^−1^, respectively, presenting superior performance compared with the previously reported results (Table S1, Supporting Information). However, CBP, m‐MoS_2_/CBP, m‐MoS_2_, and b‐MoS_2_ only release specific capacitances of 511, 517, 187, and 23 F g^−1^, respectively, at a current density of 1 A g^−1^ (Figure 3b). The Nyquist plots indicate that both MoS_2_@carbon and CBP (Figure S6d, Supporting Information) show typical electrochemical double‐layer capacitance (EDLC) behavior of the porous electrode materials.^[^ ^24^ ^]^ The cycling stability test of MoS_2_@carbon (Figure 3c) performed at 40 A g^−1^ shows that the specific capacitance remained 90.0% after 10 000 cycles, implying the excellent cyclic stability of MoS_2_@carbon. b‐MoS_2_ shows miserable electrochemical stability in the acidic electrolyte, and retains only 81.0% specific capacitance after 2000 cycles of charging‐discharging (Figure S7, Supporting Information). Probably, the carbon shell protects the ultrathin MoS_2_ nanosheets, preventing them from aggregation and addressing their problem of poor cyclic stability.

Compared with CBP, m‐MoS_2_, b‐MoS_2_, and m‐MoS_2_/CBP, MoS_2_@carbon shows much higher capacitive performance. This can be explained through the following four aspects. i) Heteroatoms in the carbon skeleton generate donor states close to the Fermi level, due to the increased charge delocalization and density. ii) Heteroatoms also enlarge the interlayer spacing of the carbon layers and increase the wettability of the electrodes. iii) The micropores in the carbon shell of MoS_2_@carbon are conducive to increasing the capacitance. vi) The ultrathin MoS_2_ nanosheets in MoS_2_@carbon comprise a large number of defects due to the polyvalent states of Mo and S, leading to the formation of more electrochemically active sites. To verify the effect of mass loading on the capacitive performance of the electrodes, we further conducted the electrochemical measurements of MoS_2_@carbon and CBP by using a mass loading of 2.0 mg cm^−2^ (Figure S8, Supporting Information). At current densities of 1, 2, 5, 10, 20, and 40 A g^−1^, the specific capacitances of MoS_2_@carbon are 1186, 1061, 917, 867, 810, and 745 F g^−1^, respectively. The specific capacitances of CBP are 443, 416, 387, 365, and 340 F g^−1^ at current densities of 1, 2, 5, 10, and 20 A g^−1^, respectively. These capacitance values are very close to those tested with a mass loading of 1.4 mg cm^−2^, featuring the excellent capacitive performance of these electrode materials.

According to the charge storage mechanism, the capacitance of a supercapacitor includes the surface‐controlled capacitance and the diffusion‐controlled contribution. We analyzed the CV curves of MoS_2_@carbon by using a reported method (details for the calculation can be found in the Supporting Information).^[^ ^29^ ^]^ The results indicate that the charge storage in MoS_2_@carbon is dominated by a surface‐controlled process (Figure S9a, Supporting Information). The diffusion‐controlled contribution reduces rapidly with the increasing scanning rate (Figure 3d). For example, at scanning rates of 50 and 5 mV s^−1^, 83.3% and 71.0% of the total capacitances of MoS_2_@carbon are contributed by the surface‐controlled process, respectively (Figure S9b,c, Supporting Information). The higher contribution of surface‐controlled capacitance is beneficial for achieving better rate performance.

We also measured the capacitive performance of MoS_2_@carbon, CBP, and b‐MoS_2_ in the 1 M KOH electrolyte, because the matching between the ion diameter of the electrolytes and the pore size of carbon materials affects the electrochemical process. Compared with the results obtained in the 1 M H_2_SO_4_ electrolyte, both MoS_2_@carbon and CBP show declined specific capacitances in the 1 M KOH electrolyte (Figure S10 and Figure S11, Supporting Information). For example, at current densities of 1.0, 2.0, 5.0, 10.0, 20.0, and 40.0 A g^−1^, MoS_2_@carbon shows specific capacitances of 963, 770, 630, 583, 545, and 506 F g^−1^, respectively. After 5000 charging–discharging cycles at a current density of 20 A g^−1^, 72.1% of the specific capacitance is retained. These results indicate that the hydronium ion is more suitable than the hydrated K^+^ for the pore size of MoS_2_@carbon and CBP. Thus, we chose the 1 M H_2_SO_4_ electrolyte for the capacitive performance measurement.

**Figure 4:**
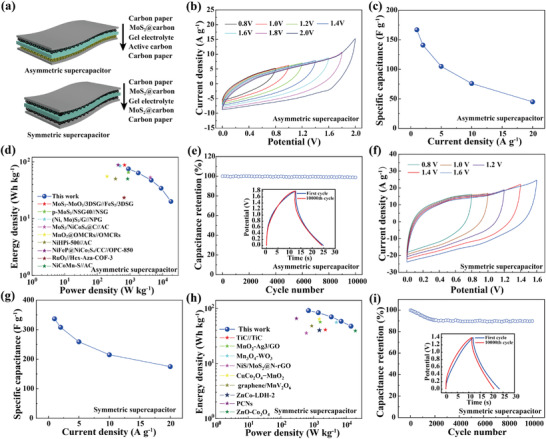
The electrochemical performances of both asymmetric and symmetric supercapacitors. a) Schematic illustration of the supercapacitor devices. CV curves of b) the asymmetric and f) the symmetric supercapacitors tested with different potential windows. Specific capacitances of c) the asymmetric and g) the symmetric supercapacitors at different current densities. Energy densities of d) the asymmetric and h) the symmetric supercapacitors at different power densities. Cycling stability of e) the asymmetric and i) the symmetric supercapacitors at current densities of 10 and 20 A g^−1^, respectively. The insets of e) and i) give the GCD curves of the 1st and 10 000th charging and discharging curves.

An asymmetric supercapacitor was constructed by sandwiching a gel electrolyte film (H_2_SO_4_‐PVA) between a MoS_2_@carbon film (positive electrode) and an activated carbon film (AC, negative electrode) (**Figure** **4**a). Based on the CV curves of MoS_2_@carbon and AC in the three‐electrode system and the asymmetric device (Figure 4b, Figure S12a,b, Supporting Information), the asymmetric supercapacitor can easily reach a voltage of 2.0 V. Considering the balance among different electrochemical properties of the device, we selected a potential window of 0–1.8 V for measurements. No obvious voltage drop is observed from the GCD curves measured at current densities ranging from 1.0 to 20.0 A g^−1^, indicating that the internal resistance of the device is very small. The specific capacitances of the asymmetric supercapacitor are calculated to be 167, 141, 105, 76, and 45 F g^−1^ at current densities of 1, 2, 5, 10, and 20 A g^−1^, respectively (Figure 4c and Figure S12c, Supporting Information). The asymmetric supercapacitor delivers an energy density of 75.1 Wh kg^−1^ at a power density of 900 W kg^−1^(Figure 4d), ranking in the first class of the recently reported asymmetric supercapacitors (Table S2, Supporting Information). The repetitive charging–discharging test features the excellent cycling stability of this asymmetric supercapacitor. As shown in Figure 4e, the asymmetric supercapacitor shows 98.1% capacitance retention after 10 000 cycles of GCD test. The Nyquist plots of the asymmetric supercapacitor change little after 10 000 cycles GCD test (Figure S12d, Supporting Information).

Then, we fabricated a symmetric supercapacitor by sandwiching a gel electrolyte film (H_2_SO_4_‐PVA) between two MoS_2_@carbon films (Figure 4a). Based on the CV curves of this symmetric supercapacitor (Figure 4f and Figure S13a, Supporting Information), we selected a potential window of 0–1.4 V to characterize the GCD curves (Figure S13b, Supporting Information). At current densities of 1.0, 2.0, 5.0, 10.0, and 20.0 A g^–1^, the specific capacitances of the symmetric supercapacitor are 377, 308, 259, 215, and 175 A g^–1^, respectively (Figure 4g). The energy density of the symmetric supercapacitor reaches 91.7 Wh kg^–1^ at a power density of 700 W kg^–1^ (Figure 4h), ranking in the first class of the recently reported symmetric supercapacitors (Table S3). After 10 000 cycles of GCD test at a current density of 20 A g^–1^, this device only exhibits a 10.1% decrease in specific capacitance and an 0.2 Ω increase in internal resistance (Figure 4i and Figure S13c, Supporting Information). The asymmetric supercapacitor shows relatively better cyclic stability than the symmetric supercapacitor. We attribute this to the activated carbon in the negative electrode of the asymmetric supercapacitor, which generally exhibits EDLC behavior and reveals excellent cyclic stability.

### 2.3 Photo‐Response of MoS_2_@Carbon

**Figure 5:**
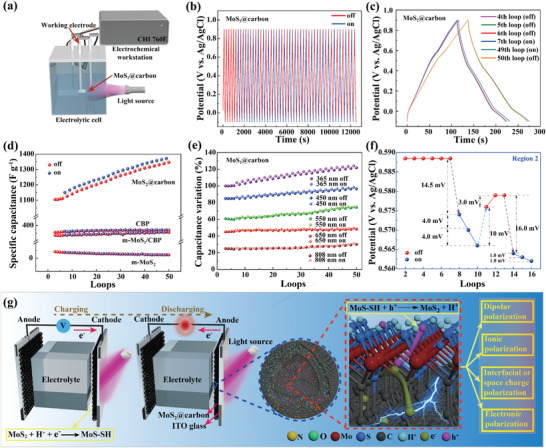
a) Schematic illustration for the photo‐response test. b) The GCD curves (10 A g^−1^, 50 loops) of MoS_2_@carbon during the switching of 365 nm UV light. c) Selected GCD curves of MoS_2_@carbon obtained with and without UV light illumination. d) The specific capacitance evolution of MoS_2_@carbon, CBP, m‐MoS_2_/CBP, and m‐MoS_2_ triggered by 365 nm UV light. e) The responsive behavior of MoS_2_@carbon triggered by light of different wavelengths. f) The change of oxidation peak at 0.5885 V in the CV curves of MoS_2_@carbon caused by 365 nm UV light illumination. g) Schematic representation for the photo‐response of MoS_2_@carbon.

The synergistic effect between the ultrathin MoS_2_ nanosheets and the carbon matrix makes MoS_2_@carbon change its capacitance under the stimulation of light. UV–vis spectrum of MoS_2_@carbon shows a broad absorption band centered at around 425 nm (Figure S14, Supporting Information). Therefore, we selected light with wavelengths of 365, 450, 550, 650, and 808 nm to stimulate MoS_2_@carbon in the three‐electrode system (**Figure** **5**a). The photo‐responsive behavior was monitored by switching the light illumination on the MoS_2_@carbon electrode during the repetitively charging‐discharging process. Both light on and light off stimulation last one charging–discharging cycle. For example, when stimulating MoS_2_@carbon through the switch of 365 nm UV light (0.08 W cm^−2^), the span of GCD curves increases and decreases reversibly (Figure 5b,c and Figure S15, Supporting Information). After each charging–discharging cycle under UV light illumination, MoS_2_@carbon exhibits an ≈3.6% (≈37 F g^−1^) increase in capacitance. Interestingly, only a ≈1.4% (≈14 F g^−1^) decrease in capacitance occurs during each charging–discharging cycle in the dark. After 50 cycles, the capacitance of MoS_2_@carbon is increased by 25.0% (Figure 5d). With the increase of light wavelength, the capacitance response amplitude of MoS_2_@carbon decreases gradually (Figure 5e). When stimulated by 450, 550, and 650 nm light (0.08 W cm^−2^), MoS_2_@carbon shows ≈2.3% (≈23 F g^−1^), ≈1.6% (≈16 F g^−1^), and ≈0.9% (≈9 F g^−1^) improvements in capacitance after each charging–discharging cycle, respectively. This stimuli‐responsive behavior was not observed when treating MoS_2_@carbon with near‐infrared light (808 nm). It can be inferred that MoS_2_@carbon undergoes both enhancement and semi‐reversible change in capacitance under light stimulation (especially 365 nm UV light).

We also characterized the photo‐responsive behavior of the control samples, including CBP, m‐MoS_2_, and m‐MoS_2_/CBP. As demonstrated in the GCD curves, their capacitances change little under the stimulation of UV light (Figure 5d), signifying the importance of core‐shell structure and the C‐Mo bond for the photo‐responsiveness of MoS_2_@carbon. To clarify this, we tracked the interaction between MoS_2_ and carbon matrix during UV light illumination. The electron paramagnetic resonance curves indicate that the UV light illumination leads to 8.2 times increase in the radical concentration of MoS_2_@carbon (Figure S16, Supporting Information). In contrast, this phenomenon was not observed in CBP and m‐MoS_2_. Apparently, efficient separation of photo‐generated electrons and holes occurs in MoS_2_@carbon.^[^ ^30^ ^]^ Probably, incorporating MoS_2_ in the carbon matrix, as well as the formation of C‐Mo bond facilitates the carrier transfer. We further monitored the CV curves evolution of MoS_2_@carbon during the switching of UV light. As shown in Figures S17 and S18, Supporting Information, the two oxidation peaks (around 0.5885 and 0.0570 V) shift close to/away from the zero potential with the switching of light, while the two reduction peaks (around 0.5780 and −0.061 V) change little. The oxidation peak at 0.5885 V was chosen as an example for tracking the reversible peak shift (Figure 5f). Taking the three loops of CV curves as one illumination cycle, the oxidation peak shifts 22.5 mV to the zero potential after the first illumination cycle. Then the oxidation peak recovers 13.0 mV during light off, but does not fully recover to the initial potential. When the illumination is applied again, the oxidation peak shifts to the zero potential by 18.0 mV, which is 4.5 mV less than that caused by the first illumination cycle. We attribute the redox peaks in the CV curves to MoS_2_, because CBP shows no redox peaks. The electrochemical redox reaction of MoS_2_ can be described by the following Equation (1):^[^ ^31^ ^]^

(1) $${\;\mathrm{MoS}}_{2}+H^{+}+e^{-}⇌\mathrm{MoS}-\mathrm{SH}$$

During the charging‐discharging process under light stimulation, MoS‐SH species can be generated, because protons inserted in the interlayers of MoS_2_ will react with Mo atoms exposed at the nanosheet edges. Based on this reaction, the mechanism for the shift of oxidation peaks in the CV curves is illustrated in Figure 5g. Upon light illumination, MoS_2_ nanosheets in the carbon matrix generate electron–hole pairs. The electrons can be easily transferred to carbon along the C‐Mo bond channel, thus efficiently suppressing the recombination of electrons and holes. At the same time, the holes will be captured by MoS‐SH, thus promoting the oxidation reaction and lowing the oxidation reaction potentials. In general, the capacitance of materials depends on the polarization, including dipolar polarization, ionic polarization, interfacial or spatial charge polarization, and electronic polarization.^[^ ^32^ ^]^ Accordingly, the light‐triggered capacitance evolution of MoS_2_@carbon can be attributed to two factors. First, the photo‐generated carriers can effectively enhance dipolar polarization, ion polarization, and electronic polarization. Second, electrons and holes accumulated in the carbon matrix and MoS_2_ nanosheets improve the interfacial and spatial charge polarization.

**Figure 6:**
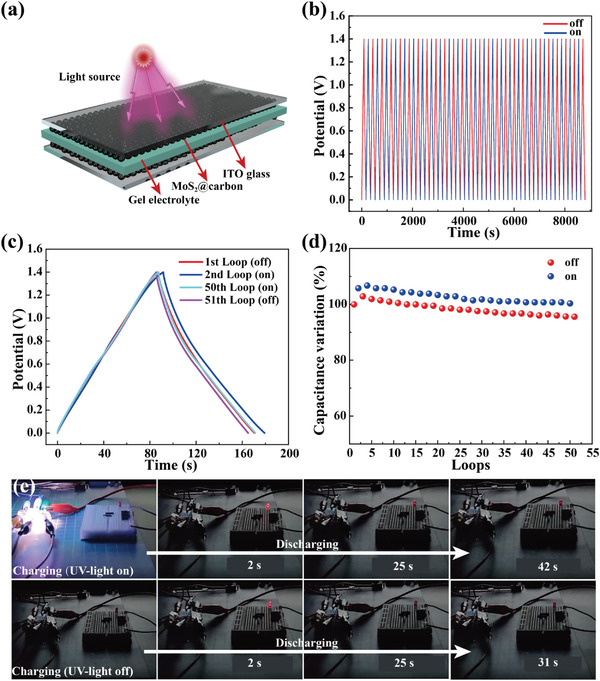
a) Schematic illustration of PRSC. b) The GCD curves (current density: 5 A g^−1^, 50 loops) of PRSC during the switching of 365 nm UV light illumination. c) The comparison of GCD curves at the beginning and end of illumination. d) Capacitance evolution of PRSC measured with UV light on and off. e) The luminescence duration comparison of a diode powered by PRSC charged with and without UV light illumination.

Finally, we assembled a photo‐responsive symmetric supercapacitor device (PRSC) by sandwiching a gel electrolyte film of H_2_SO_4_‐PVA between two electrodes, which were constructed by coating MoS_2_@carbon on the surface of indium tin oxide glass (**Figure** **6**a). The photo‐responsive behavior of PRSC was monitored by using the GCD test (current density: 5 A g^–1^) under 365 nm (0.08 W cm^−2^) UV light illumination (Figure 6b,c). The corresponding capacitance evolution is given in Figure 6d. PRSC exhibits a ≈4.50% (≈13.9 F g^−1^) capacitance increase in each charging–discharging cycle with UV light treatment. Under darkness treatment, each charging‐discharging cycle induces a ≈4.59% (≈14.2 F g^−1^) drop in capacitance. These results imply that MoS_2_@carbon show almost full‐reversible photo‐responsive behavior in the symmetric supercapacitor. Due to the unique photo‐responsive property, the energy supply process of PRSC can be easily manipulated by light. We directly tracked this smart behavior by lighting a diode with PRSC. As shown in Figure 6e and Movie S1, Supporting Information, PRSC charged in darkness can only keep the diode lighting for 31 s. In comparison, PRSC charged under UV light illumination lights the diode for 42 s, which is prolonged by 35%, signifying that PRSC is a self‐powered photo‐responsive actuator.

## 3 Conclusion

In summary, by using BPs as the coordination platform, we have developed an in situ approach to generate ultrathin MoS_2_ nanosheets in the carbon matrix, thus forming a MoS_2_@carbon core‐shell structure. The composition and structure features make MoS_2_@carbon exhibit outstanding capacitive performance and photo‐responsiveness. First, due to the doping of B, N, and O in the carbon matrix, as well as the microporous structure, MoS_2_@carbon shows a high specific capacitance of 1302 F g^−1^ (current density:1 A g^−1^) in the three‐electrode system. Capacitance retention of 90.0% is achieved after 10 000 charging–discharging cycles at a current density of 40 A g^−1^. The asymmetric supercapacitor displays an energy density of 75.1 Wh kg^−1^ at a power density of 900 W kg^−1^. In the case of the symmetric supercapacitor, the energy density reaches 91.7 Wh kg^−1^ at a power density of 700 W kg^−1^. Second, the ultrathin MoS_2_ nanosheets are sensitive to light illumination, while the core‐shell structure and C‐Mo bond facilitate the energy and mass exchanges between MoS_2_ nanosheets and carbon matrix. Thereby, both the transfer of photo‐generated electrons to the carbon matrix and the capture of holes in MoS_2_ nanosheets are enhanced, causing the capacitance evolution of MoS_2_@carbon. The three‐electrode system and the symmetric supercapacitor exhibit ≈3.6% (≈37 F g^−1^) and ≈4.5% (≈13.9 F g^−1^) improvements in capacitance during each charging–discharging cycle, respectively, under the stimulation of 365 nm UV light illumination (0.08 W cm^−2^). Especially in the symmetric supercapacitor, the photo‐response of MoS_2_@carbon has good stability and reversibility. By combining the advantages of large capacitance, high energy density, and excellent photo‐responsiveness, MoS_2_@carbon would be of great interest in constructing self‐powered smart devices. Additionally, the use of BPs as the coordination platform may provide a highly adaptable strategy for the design and synthesis of multi‐functional energy storage materials.

## Conflict of Interest

The authors declare no conflict of interest.

## Supporting information

Supporting Information

Supplemental Movie 1

## Data Availability

The data that support the findings of this study are available in the supplementary material of this article.
