# Supplementary material for: In Situ Generation of Ultrathin MoS2 Nanosheets in Carbon Matrix for High Energy Density Photo‐Responsive Supercapacitors
Source: Adv Sci (Weinh). 2022 Jul 7;9(24):2201685. doi: 10.1002/advs.202201685 (PMC9404387; doi:10.1002/advs.202201685)
Supplement: Supplementary file 1 — Supporting Information [file ADVS-9-2201685-s001.pdf]

Supporting Information

In-situ Generation of Ultrathin MoS_2 Nanosheets in Carbon Matrix for High Energy Density Photo-responsive Supercapacitors

Zhenbin Tang[1], Juguo Dai[1], Wenkang Wei[1], Zhi Gao[1], Zhixuan Liang[1], Chenzhi Wu[1], Birong Zeng[1,2], Yiting Xu[1,2], Guorong Chen[1,2], Weiang Luo[1,2], Conghui Yuan[1,2], Lizong Dai [1,2]**

[1] College of Materials, Xiamen University, Xiamen 361005, People's Republic of China.
[2] Fujian Provincial Key Laboratory of Fire Retardant Materials, Xiamen University, Xiamen 361005, People's Republic of China.
Corresponding Author: *yuanch@xmu.edu.cn, *lzdai@xmu.edu.cn

1. Experimental section

1.1 Material

3, 4-Dihydroxybenzaldehyde, 4-formylphenylboronic acid, tris (4-aminophenyl) amine, and ammonium tetrathiomolybdate were purchased from J&K Chemical. Monolayer MoS_2 was supplied by Shanghai Xianding Biotechnology. Potassium hydroxide (KOH), concentrated sulfuric acid (H_2SO_4, 98%) and conventional solvents were purchased from Shanghai Chemical Reagent Industry. Polyvinyl alcohol (PVA) and activated carbon (AC) were supplied by Aladdin. The Indium Tin Oxide (ITO) glass was purchased from Foshan Shi Yuan Jing Mei Glass Company Limited. All chemicals were used as received without further purification. Catechol monomer TAC and boronic monomer TAB were synthesized according to our previous work.[1]

1.2 Synthesis of BP, MoS_4^{2-}-BP and MoS_4^{2-}-TAC

Synthesis of BP: Typically, 50.0 mg (0.0769 mmol) of TAC and 52.8 mg (0.0769 mmol) of TAB were separately dissolved in 35.0 mL of ethanol. The TAC solution was added dropwise into the TAB solution under vigorous stirring. After reacting at room temperature overnight, red product of spherical BP was collected by centrifugation, followed by washing with ethanol for three times, and drying in vacuum at 60 °C.

Synthesis of MoS_4^2-BP: $(NH_4)_2MoS_4$ (54.0 mg, 0.2075 mmol) was dissolved in a mixed

solution containing 0.4 mL of *N, N*-dimethylformamide and 60 mL of methanol. Then, BP (100.0 mg) was dispersed in the above $(NH_4)_2MoS_4$ solution under sonication. After stirring for 2 h, red product of MoS_4^{2-}-BP was collected by centrifugation. The as obtained MoS_4^{2-}-BP was washed three times with anhydrous methanol, and dried in vacuum at 60 °C.

Synthesis of MoS_4^{2-}-TAC: $(NH_4)_2MoS_4$ (10.0 mg, 0.0384 mmol) was dissolved in a mixed solution containing 0.1 mL of *N, N*-dimethylformamide and 15.0 mL of methanol. After introducing TAC (49.94 mg, 0.0768 mmol) into the $(NH_4)_2MoS_4$ solution, the reaction was allowed at room temperature for 2 h under stirring. Finally, black precipitate (MoS_4^{2-}-TAC) was obtained after centrifugation, washing and drying in vacuum at 60 °C.

1.3 Preparation of MoS_2@carbon

MoS_2@carbon was obtained by a two-step thermolysis process. MoS_4^{2-}-BP was firstly dried at 100 °C for 1 h under the protection of 5% H_2/95%N_2 atmosphere, and then heated to 500°C at a heating rate of 5 °C/min. This temperature was maintained for 1 h. Then, the sample was heated to 950 °C at a heating rate of 5 °C/min under the protection of Ar atmosphere. After 0.5 h reaction, the system was naturally cooled to room temperature. Control samples of CBP and b-MoS_2 were prepared by sintering BP and $(NH_4)_2MoS_4$ under the same condition.

1.4 Characterization

The crystalline structure of the samples was characterized by X-ray diffraction (Bruker-AXS D8-A25) using Cu Kα radiation (λ=0.15141 nm). The elemental composition of the samples were analyzed by using PHI Quantum-2000 photo-electron spectrometer (Al Kα with 1486.6 eV). The morphology of the samples were observed by using the transmission electron microscopy (JEM 2100) with an acceleration voltage of 200 kV. The elemental energy-dispersive X-ray spectroscopy (EDX) mapping was conducted on a FEI Talos F200S instrument. Raman spectra were tested on an Xplora Raman spectrophotometer (excitation wavelength: 532 nm). The Brunauer-Emmett-Teller (BET) surface area and pore size distribution of the samples were characterized using an ASAP 2460 system (temperature: 77 K). The electron spin resonance (EPR) spectra were acquired on Bruker EMX-10/12 (temperature: 300 K). The UV-vis spectra were tested on Shimadzu UV-2550. Fourier transform infrared (FT-IR) spectra were characterized on Nicolet Avatar 360. The Mo content of MoS_2@carbon was determined by inductively coupled plasma-atomic emission spectroscopy (ICP-AES) with a Perkin Elmer Nexion 300. Perfectlight PLS-

SEX300D/300DUVA Xenon lamp light source assembled with 365, 450, 550, 650, 808 nm filters was adopted in the photo-response characterization.

1.5. Calculation of the average size of MoS_2@carbon, CBP, and BP

The average sizes of MoS_2@carbon, CBP, and BP were calculated from TEM images by Digital Micrograph (statistical magnitude is 100) with **Equation S(1)**:

$$D=\frac{\sum_i^{100} d_i}{100} \tag{1}$$

where D is the average size of MoS_2@carbon, CBP, and BP; d_i is the particle size measured from TEM image; i is the sequence number of selected particles.

The standard deviations of MoS_2@carbon, CBP, and BP were calculated by **Equation S(2)**:

$$s=\sqrt{\frac{\sum_i^{100} (d_i-D)^2}{99}} \tag{2}$$

where s is the standard deviation of MoS_2@carbon, CBP, and BP; D is the average size of MoS_2@carbon, CBP, and BP; d_i is the particle size measured by TEM image; i is the sequence number of selected particles.

2. Electrochemical measurement

Electrochemical workstation (CHI 760E) was applied for the electrochemical measurements. In the typical three-electrode system, a reference electrode Ag/AgCl, a counter electrode Pt foil, and a working electrode glassy carbon were used. 1 M H_2SO_4 and 1 M KOH aqueous solutions were used as acidic and alkaline electrolytes, respectively. The working electrode was fabricated by mixing active materials (5 mg), ultrapure water (65 μL), ethanol (25 μL) and 5 wt% Nafion solution (10 μL, DuPont, USA) (the mass of active materials is 1.4 mg cm^{-2}). The potential of the reference electrode Ag/AgCl was calibrated to the reversible hydrogen electrode (RHE) by **Equation S(3)**:

$$E\ (RHE)=E\ (Ag/AgCl)+0.1989\ V+0.059\times pH \tag{3}$$

All potentials of the three-electrode system were referred to RHE.

The symmetric and asymmetric supercapacitors are constructed using the two-electrode system. The gel electrolyte was prepared by dissolving 5.0 g PVA in 30 mL of 1 M H_2SO_4

aqueous solution. To construct the symmetric supercapacitor, the MoS_2@carbon slurry was coated on carbon papers to act as both positive and negative electrodes (mass loading: 2 mg cm^{-2}). When fabricating the photo-responsive device PRSC, MoS_2@carbon slurry was coated on the ITO glasses.

In the asymmetric supercapacitor, MoS_2@carbon slurry was coated on the carbon paper to form the positive electrode (mass loading: 2 mg cm^{-2}), and activated carbon slurry was coated on the carbon paper to serve as the negative electrode. The mass of active carbon slurry is determined by **Equation S(4)**:

$$\frac{m_+}{m_-}=\frac{C_- \times \Delta V_-}{C_+ \times \Delta V_+} \tag{4}$$

Where m (g) is the mass of electroactive materials, C (F g^{-1}) is the specific capacitance, ΔV (V) is the voltage range.

The specific capacitance (C, F g^{-1}) of the samples was determined by the discharging portion of the GCD curves, and calculated by **Equation S(5)**:

$$C=\frac{I \times \Delta t}{m \times \Delta V} \tag{5}$$

Where, I (A) is the discharging current, Δt (s) is the discharging time, and ΔV (V) is the voltage. In the three-electrode system, m (g) is the mass of the active material coated on the working electrode. In the two-electrode system (symmetric and asymmetric supercapacitors), m (g) represents the mass of the active material in the positive electrode and negative electrode.

The energy density (E, Wh kg^{-1}) and power density (P, W kg^{-1}) of the symmetric and asymmetric supercapacitors were estimated by **Equation S(6)** and **(7)**, respectively:

$$E=\frac{C \times \Delta V^2}{7.2} \tag{6}$$

and

$$P=\frac{3600E}{\Delta t} \tag{7}$$

Where, C (F g^{-1}) represents the specific capacitance of the symmetric and asymmetric supercapacitors, ΔV (V) represents the voltage, Δt (s) represents the discharging time.

The CV curves (three-electrode system) of MoS_2@carbon at various scanning rates were used to determine the charge storage mechanism. Peak currents (i, A) were obtained at different scanning rates (v, mV/s) from the CV curves. The charge storage mechanism of the electrode materials can be predicted by calculating b using **Equation S(8)** and **(9)**:[2]

$$i=av^b \tag{8}$$

$$Log\ i=b\ Log\ v+Log\ a \tag{9}$$

where a, b are constants and b is generally in the range of 0.5-1. b = 0.5 indicates the diffusion-controlled process, while b=1.0 indicates the surface-controlled process. If b is in the range of 0.5-1, the electrode material exhibits both diffusion-controlled process and the surface-controlled process.

The contribution of the surface-controlled capacitance and diffusion-controlled capacitance to the total capacitance was calculated by **Equation S(10)** and **(11)**:[3]

$$I_{(V)}=k_1v+k_2v^{1/2} \tag{10}$$

$$I_{(V)}/v^{1/2}=k_1\ v^{1/2}+k_2 \tag{11}$$

Where, $I_{(V)}$ is the current at a fixed voltage, v is the scanning rate, k_1 and k_2 are constants. Towards a specific voltage, k_1 is obtained by linearly fitting of $i_{(V)}/ v^{1/2}$ and $v^{1/2}$. Different specific voltages correspond to different fitted k_1. Simultaneously, $k_1\ v$ and $k_2\ v^{1/2}$ correspond to the current contributions from the surface capacitive effect and the diffusion-controlled intercalation process, respectively. The surface-controlled capacitance also reflects the rate performance of the electrode materials as well as the surface charge storage capability.

3. Photo-response measurement of MoS_2@carbon

The light-triggered capacitance evolution of MoS_2@carbon was first investigated in the three-electrode system, in which Ag/AgCl, Pt foil, glassy carbon, and 1 M H_2SO_4 were used as the reference electrode, counter electrode, working electrode, and electrolyte, respectively. The working electrode was fabricated according to the above-described method in the section of electrochemical measurement. The light illumination was controlled by Perfectlight PLS-SEX300D/300DUVA Xenon lamp. During the switching of the light, the GCD and CV curves were acquired. Both light on and light off treatments maintain for a whole GCD or CV test cycle.

The photo-responsive device PRSC is a typical two-electrode planar symmetric supercapacitor. In this device, the gel electrolyte was prepared by dissolving 5.0 g PVA in 30 mL of 1 M H_2SO_4 aqueous solution. The electrodes were fabricated by coating MoS_2@carbon slurry on the ITO glasses. The photo-response of the PRSC was characterized using the same method applied in the three-electrode system.

4. Results and discussion

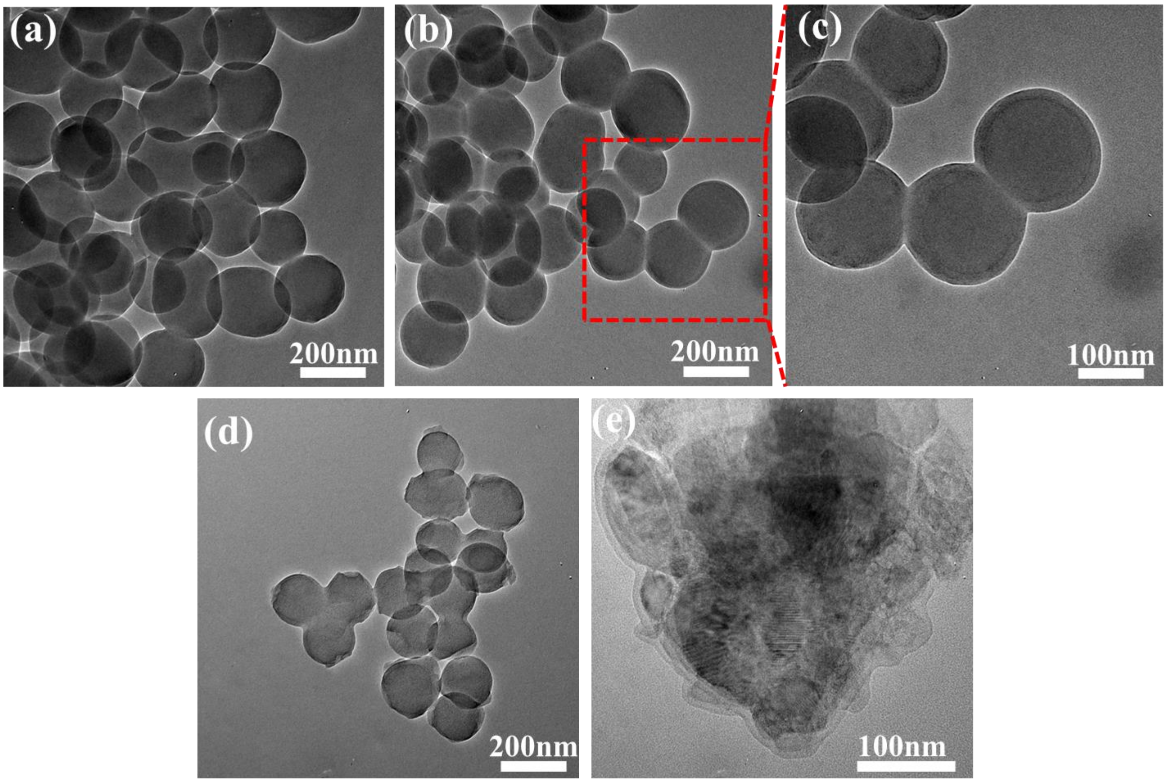

Figure S1. TEM images of (a) BP, (b, c) MoS_4^{2-}-BP, (d) CBP, and (e) b-MoS_2.

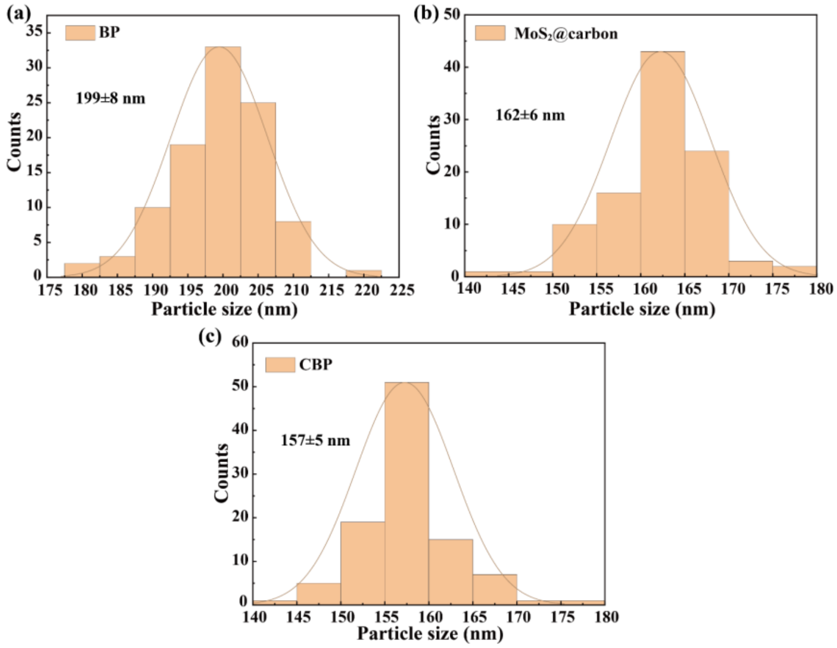

Figure S2. Size distribution of (a) BP, (b) MoS_2@carbon, and (c) CBP.

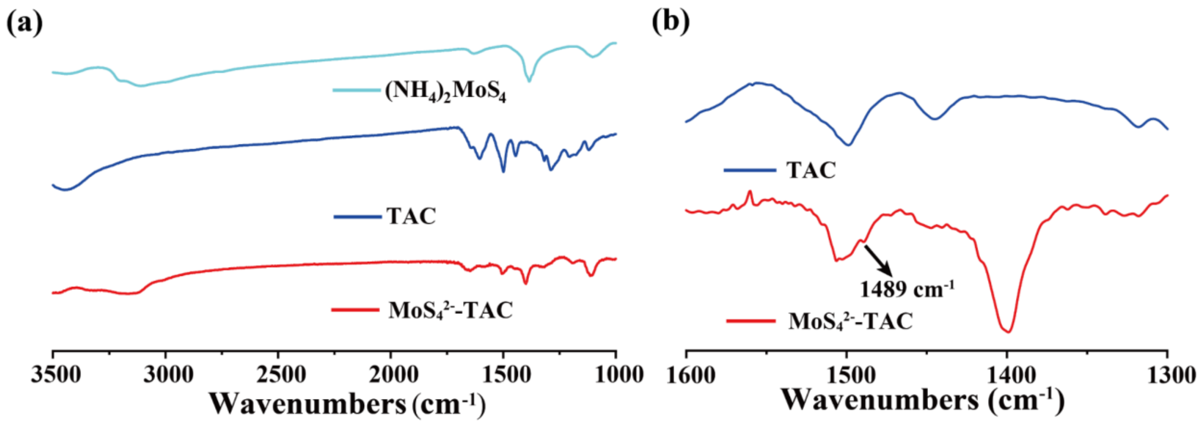

Figure S3. (a) FT-IR spectra of TAC, $(NH_4)_2MoS_4$, and MoS_4^{2-}-TAC. (b) Enlarged area of the FT-IR spectra of TAC and MoS_4^{2-}-TAC (a new peak at 1489 cm^{-1} indicates the coordination between TAC and MoS_4^{2-}).

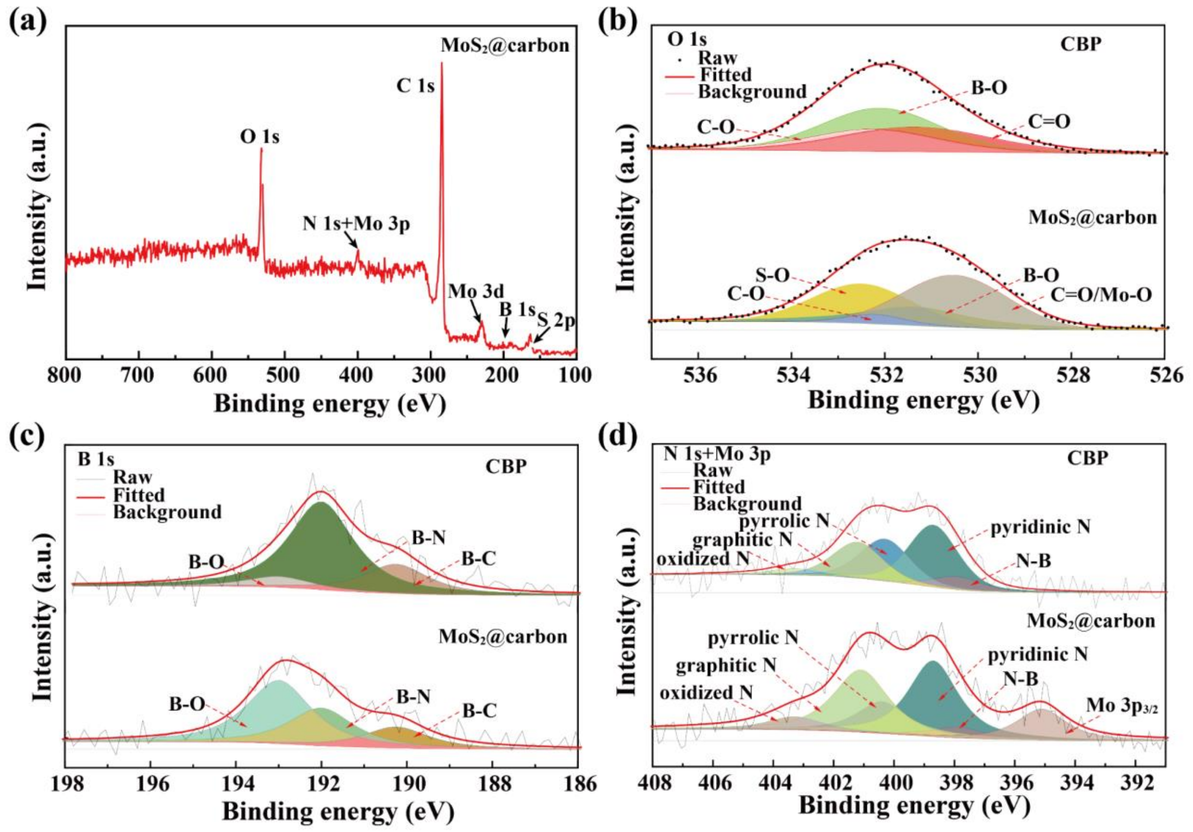

Figure S4. (a) XPS survey spectra of MoS_2@carbon. High-resolution XPS spectra of (b) O 1s, (c) B 1s, and (d) N 1s+Mo 3p for MoS_2@carbon and CBP.

The O 1s spectrum of MoS_2@carbon can be deconvoluted into four binding energies centered at 532.2, 532.1 531.5, and 530.5 eV, which are assigned to C-O, S-O, B-O, and C=O/Mo-O, respectively.[4] The B 1s signal of MoS_2@carbon can be curve-fitted into three

peaks with binding energies of 193.0, 192.0, and 190.3 eV, corresponding to B-O, B-N and B-C, respectively.[5] In the N 1s + Mo 3p spectrum of MoS_2@carbon, the peaks at 395.0, 397.9, 401.1, 403.3, 398.7, and 400.4 eV are ascribed to Mo $3p_{3/2}$, N-B bond, graphitic N, oxidized N, pyridinic N, and pyrrolic N, respectively.[6]

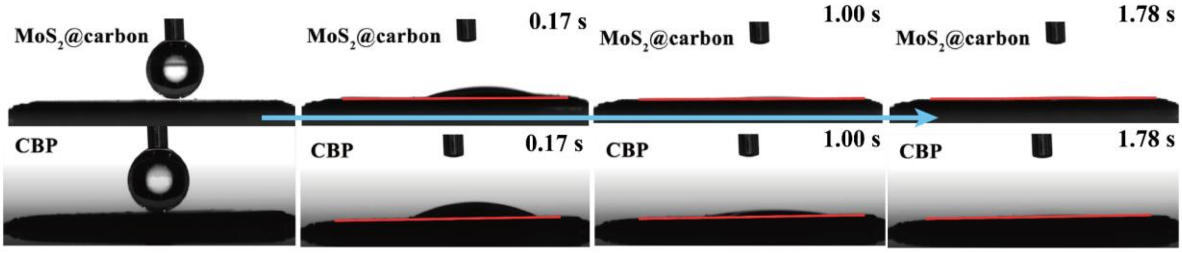

Figure S5. The contact angle of water on the surface of MoS_2@carbon and CBP.

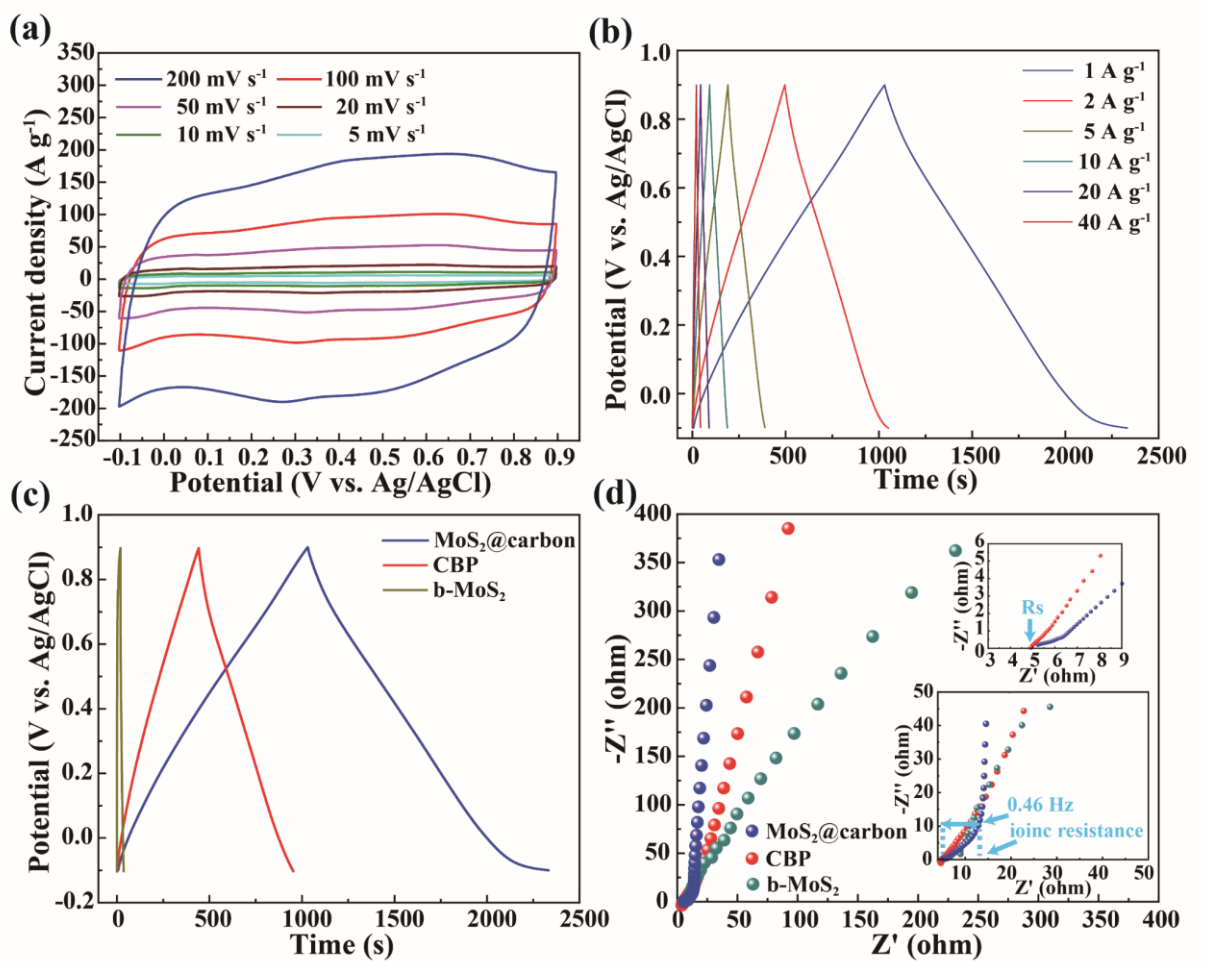

Figure S6. The electrochemical performances of the samples in the three-electrode system (1 M H_2SO_4 electrolyte). (a) The CV curves of MoS_2@carbon at different scanning rates. (b) The GCD curves of MoS_2@carbon at different current densities. (c) GCD curves of MoS_2@carbon, CBP, and b-MoS_2 at the current density of 1 A g^{-1}. (d) Nyquist plots of MoS_2@carbon, CBP, and b-MoS_2.

In the Nyquist plots, R_s is the intersecting point with Z' axis and represents the bulk resistance, and the arc in the high frequency region represents the ionic resistance. The R_s values of MoS_2@carbon, CBP, and b-MoS_2 are 5.23, 4.95, and 8.95 Ω, respectively. The ionic resistance values of MoS_2@carbon and CBP are 7.89 and 8.14 Ω, respectively. This means that MoS_2@carbon and CBP show similar resistance.

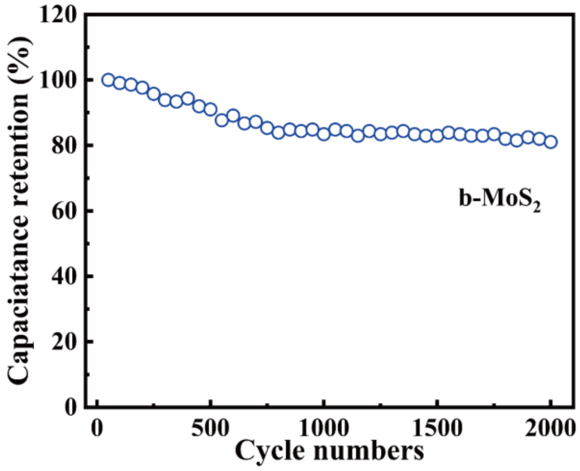

Figure S7. Cyclic stability of b-MoS_2 in the 1 M H_2SO_4 electrolyte at a current density of 1 A g^{-1}.

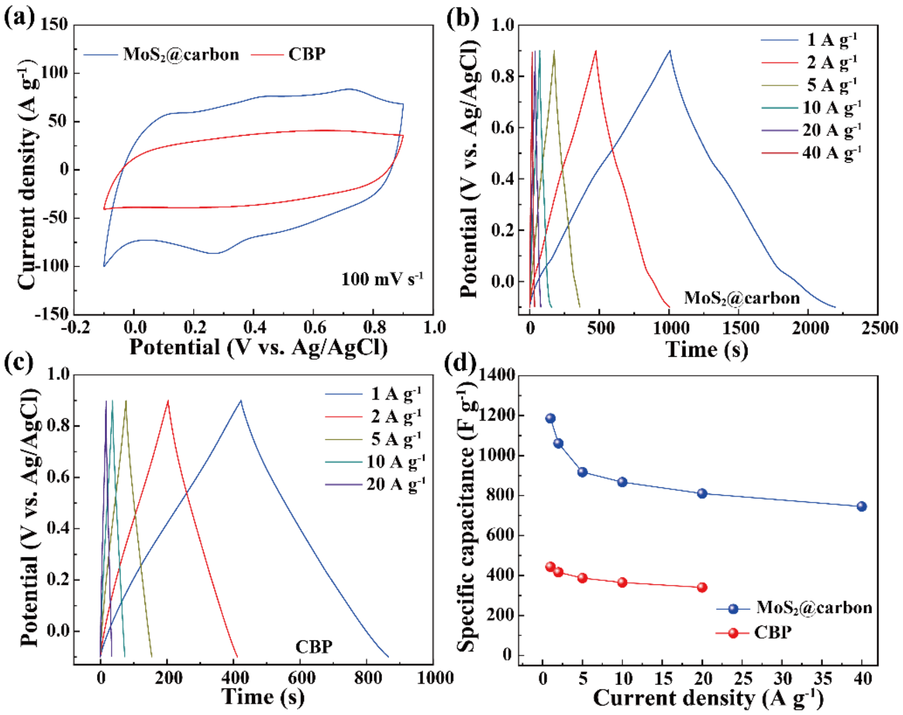

Figure S8. Three-electrode system electrochemical tests with a mass loading of 2.0 mg cm^{-2} in the electrodes. (a) CV curves of MoS_2@carbon and CBP at a scan rate of 100 mV s^{-1}. GCD curves of (b) MoS_2@carbon and (c) CBP. (d) The specific capacitances of MoS_2@carbon and CBP.

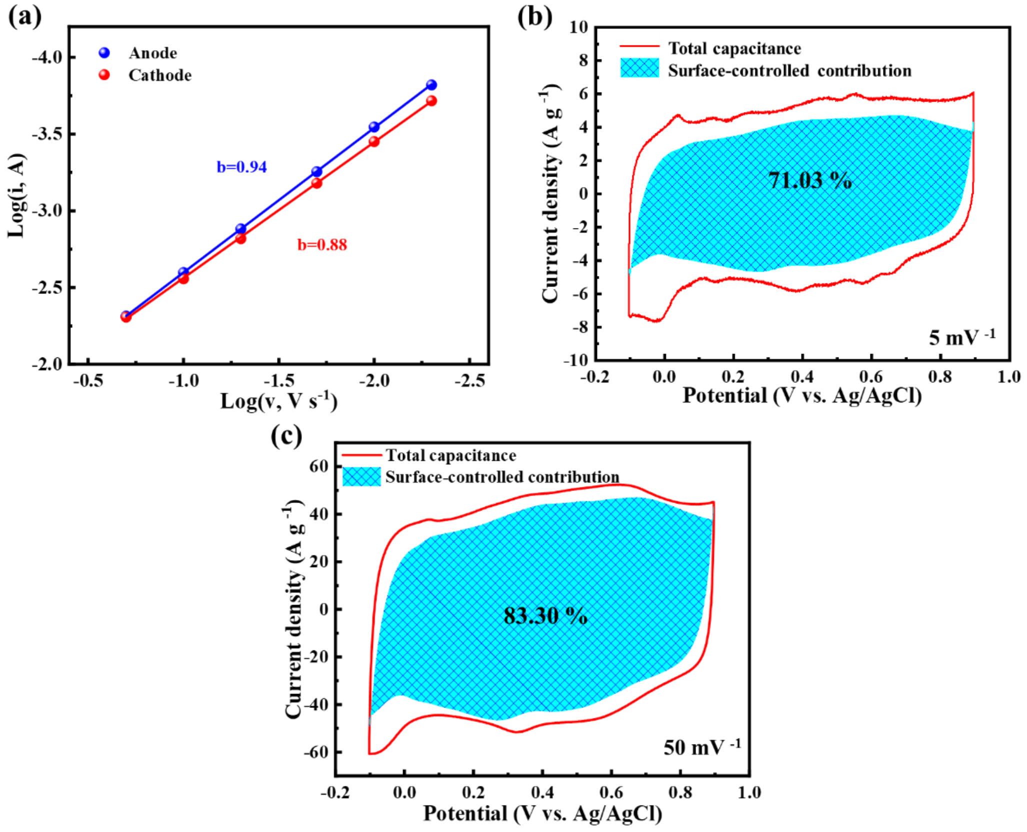

Figure S9. (a) Plots for Log *i* versus Log *v* of MoS_2@carbon, where *i* represents the peak currents at the anode and cathode, *v* is the scanning rate (three-electrode system, electrolyte: 1 M H_2SO_4). Surface-controlled capacitance contribution at scanning rates of (b) 5 mV s^{-1} and (c) 50 mV s^{-1}.

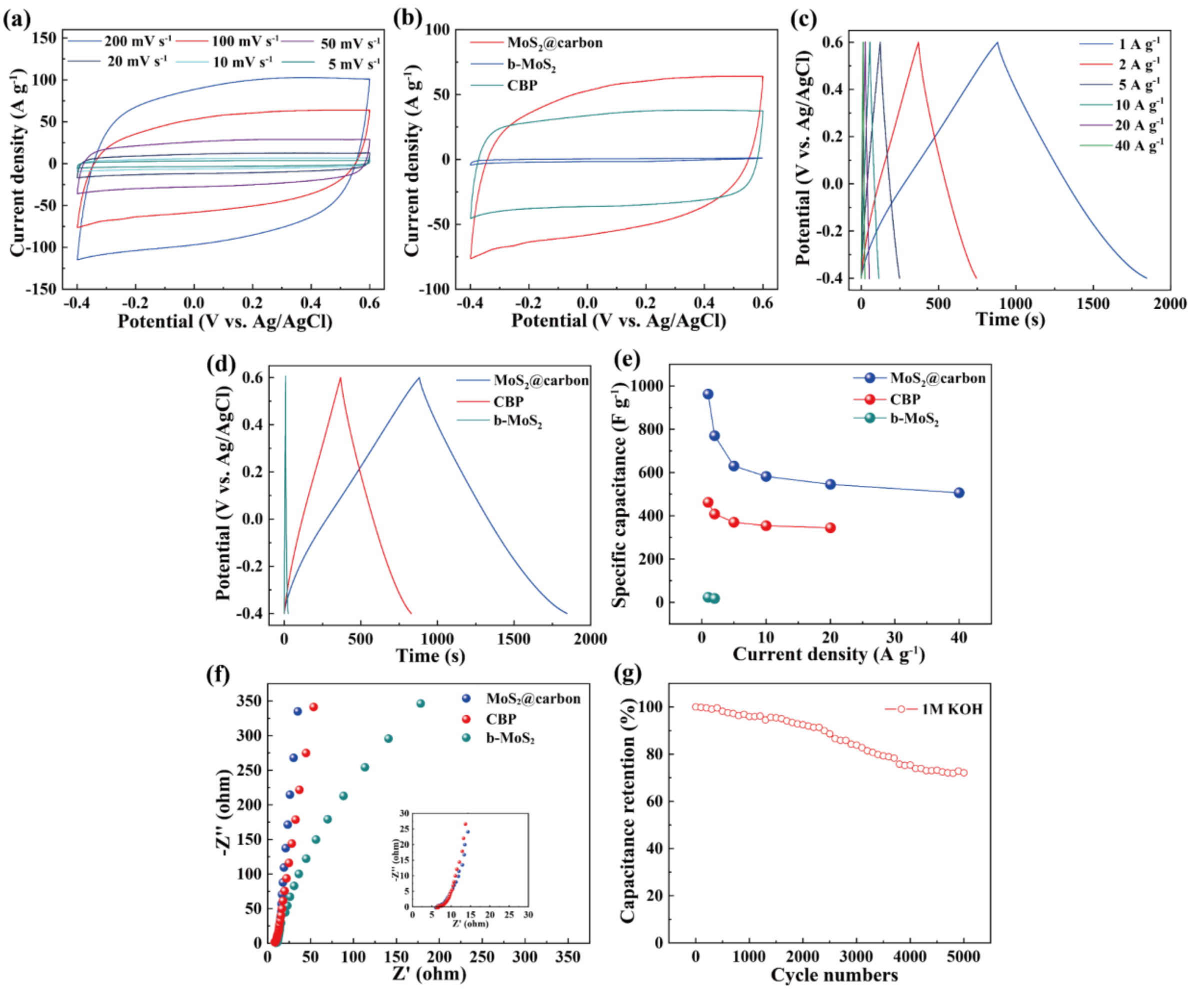

Figure S10. The electrochemical performances of the samples in the three-electrode system using 1 M KOH as the electrolyte. (a) CV curves and (c) GCD curves of MoS_2@carbon at different current densities. (b) CV curves and (d) GCD curves of different samples measured at the scanning rate of 100 mV s^{-1} and the current density of 1 A g^{-1}. (e) The capacitive performance versus current density. (f) Nyquist plots for MoS_2@carbon, CBP, and b-MoS_2. (g) Cyclic stability of MoS_2@carbon at the current density of 20 A g^{-1}.

In 1 M KOH aqueous solution, the CV curves of MoS_2@carbon present a typical rectangle shape without redox peaks, indicating that MoS_2@carbon mainly exhibits EDLC behavior in the alkaline condition. At current densities of 1.0, 2.0, 5.0, 10.0, 20.0, and 40.0 A g^{-1}, MoS_2@carbon shows specific capacitances of 963, 770, 630, 582, 545, and 507 F g^{-1}, respectively. In comparison, the specific capacitances of CBP and b-MoS_2 are 462 and 23.3 F g^{-1}, respectively, at a current density of 1 A g^{-1}. In the Nyquist plots, the slope of MoS_2@carbon is higher than those of CBP and b-MoS_2 at low frequencies, indicating that MoS_2@carbon shows better capacitive performance.

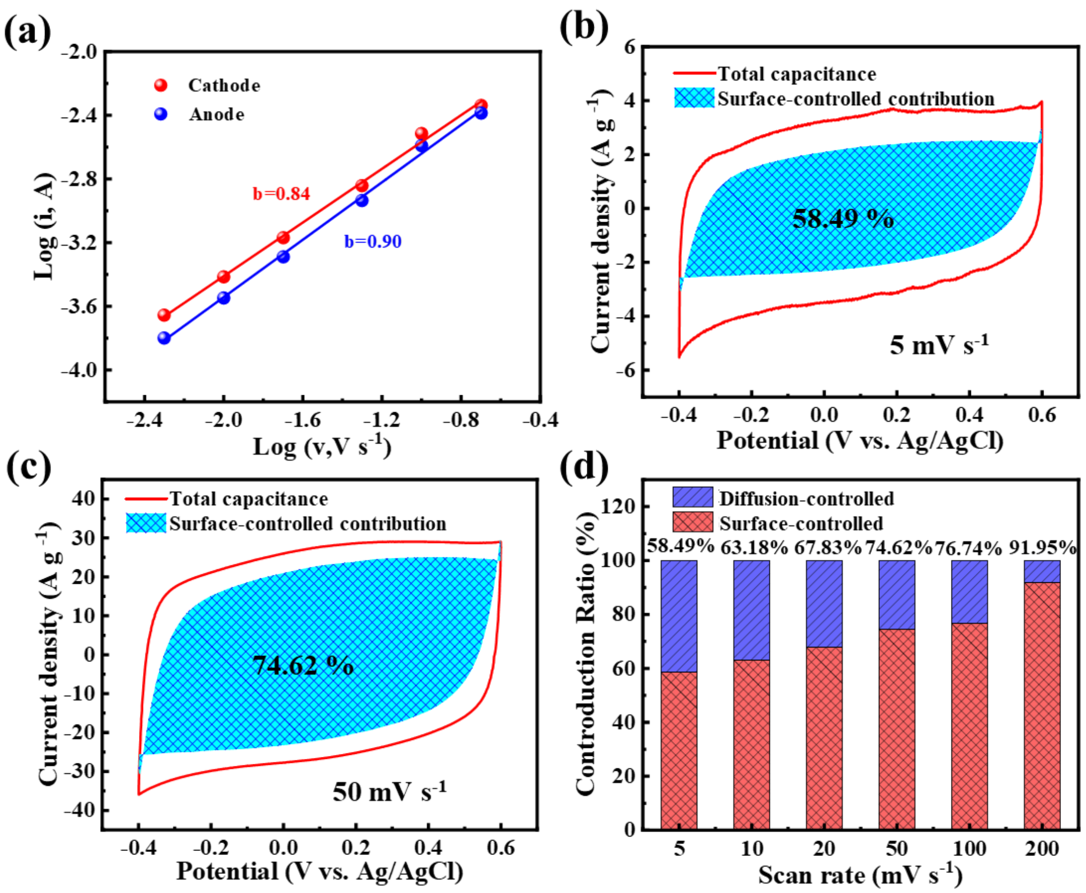

Figure S11. (a) Plots for Log *i* versus Log *v* of MoS_2@carbon, where *i* represents the peak currents at the anode and cathode, *v* is the scanning rate (three-electrode system, electrolyte: 1 M KOH). Contribution of surface-controlled capacitance at scanning rates of (b) 5 mV s^{-1} and (c) 50 mV s^{-1}. (d) Diagram for the contributions of diffusion-controlled and surface-controlled capacitances to total capacitances at different scanning rates.

From **Figure S11a**, the values of *b* are calculated to be 0.90 for the anode and 0.84 for the cathode, implying that the surface-controlled process is dominant for the charge storage. Moreover, 74.62 % and 58.94 % of the total capacitances are contributed by the surface-controlled process at 50 and 5 mV s^{-1}, respectively (**Figure S11b-d**). This result implies that the contribution of diffusion-controlled capacitance drops rapidly with the increasing scanning rate.

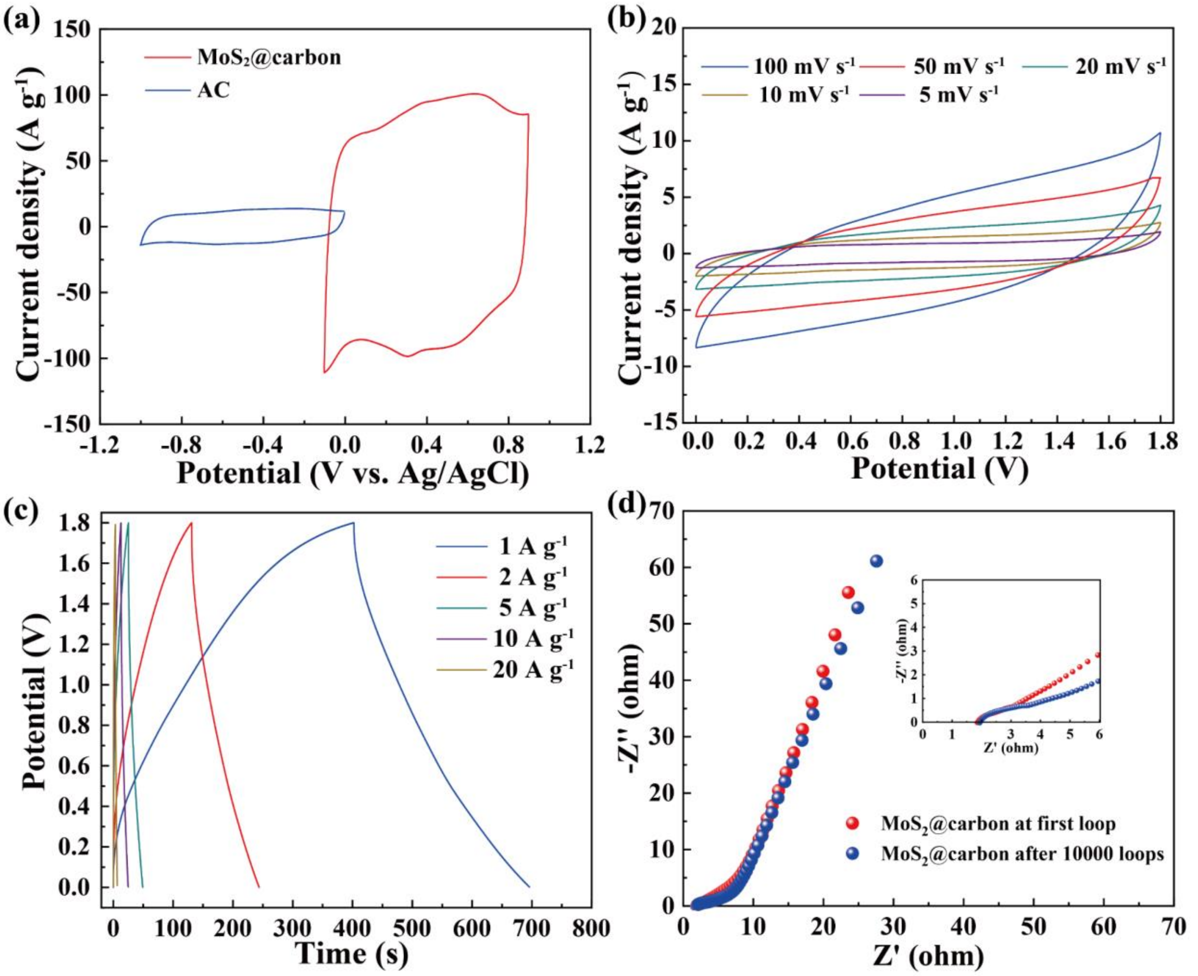

Figure S12. (a) CV curves (scan rate: 100 mV s^{-1}) of MoS_2@carbon and AC in the three-electrode system. (b) CV curves at different scan rates, (c) GCD curves at different current densities, and (d) Nyquist plots at 1st, 10000th cycles of the asymmetric supercapacitor.

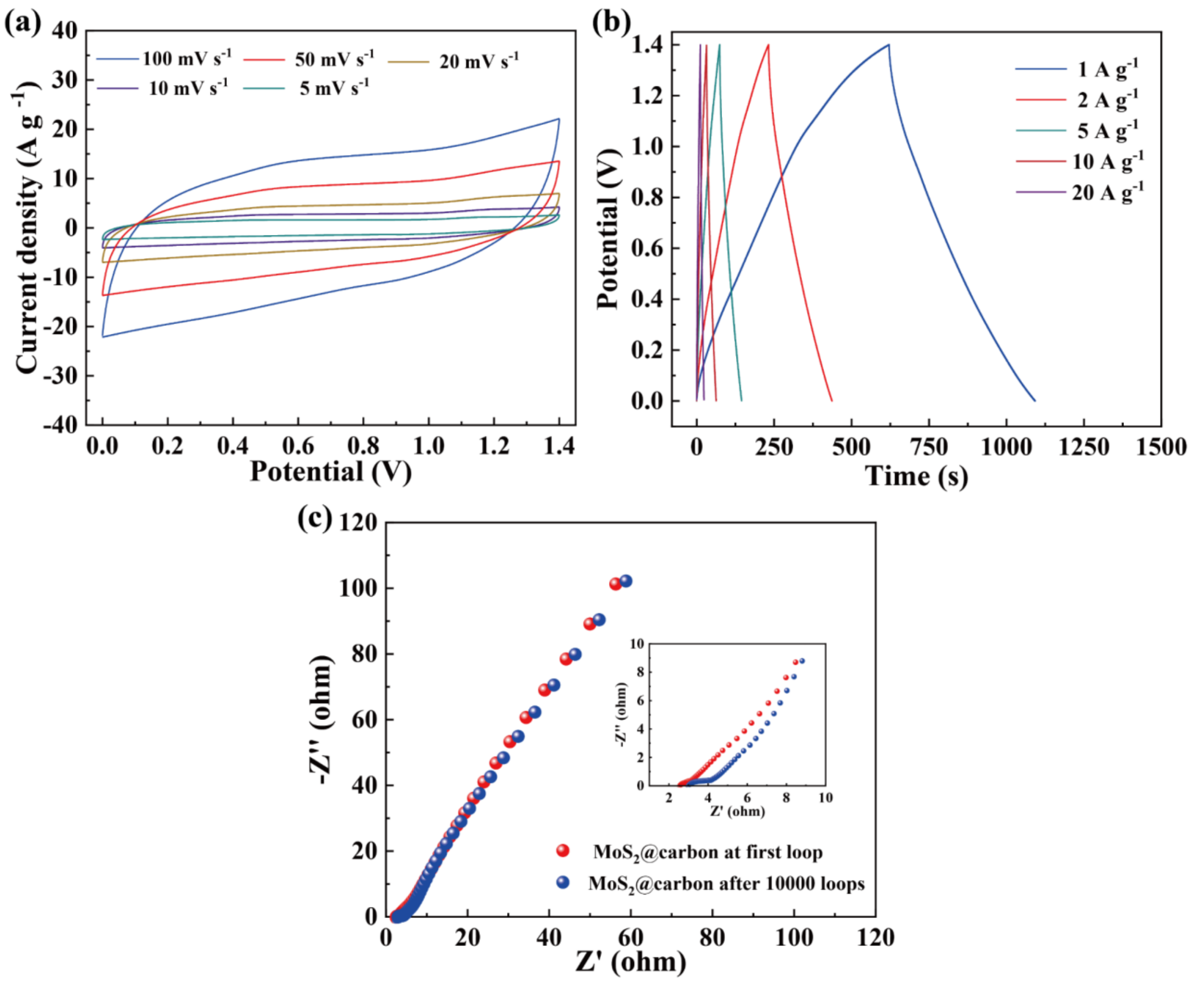

Figure S13. Electrochemical performance of the symmetric supercapacitor. (a) CV curves at different scan rates, (b) GCD curves at different current densities, and (c) Nyquist plots at 1st, 10000th cycles.

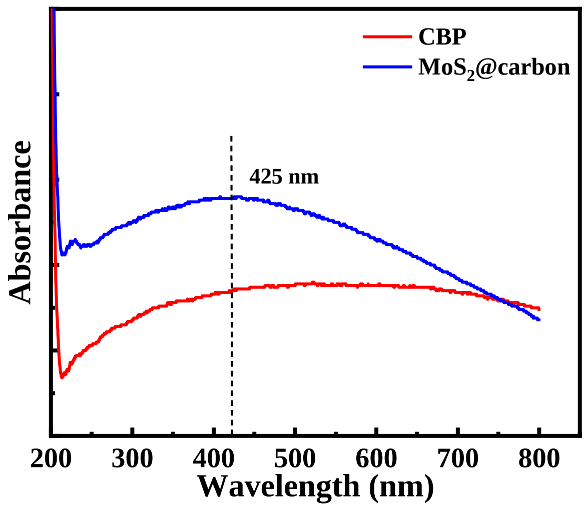

Figure S14. UV-vis spectra of CBP and MoS_2@carbon.

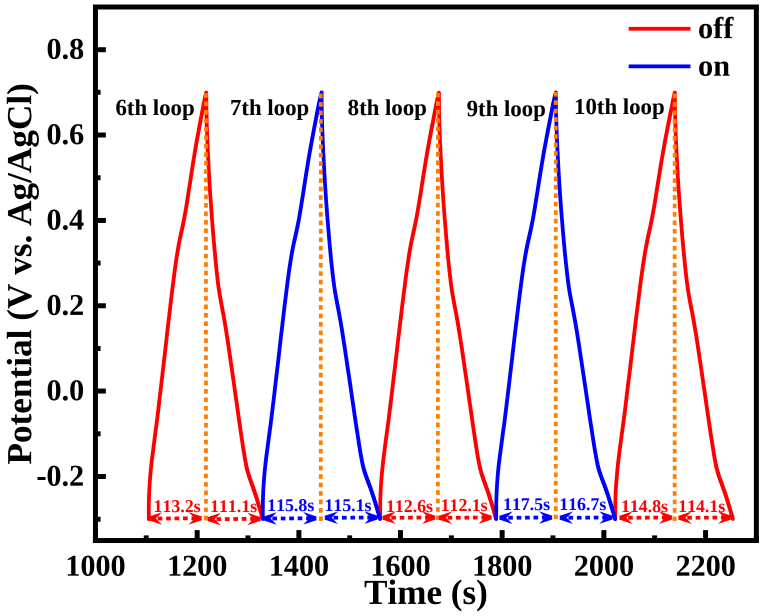

Figure S15. Charging-discharging duration times in the GCD curves of MoS_2@carbon (from 6 to 10 loops). These curves were measured when alternately treating the testing system with 365 nm UV light illumination and darkness.

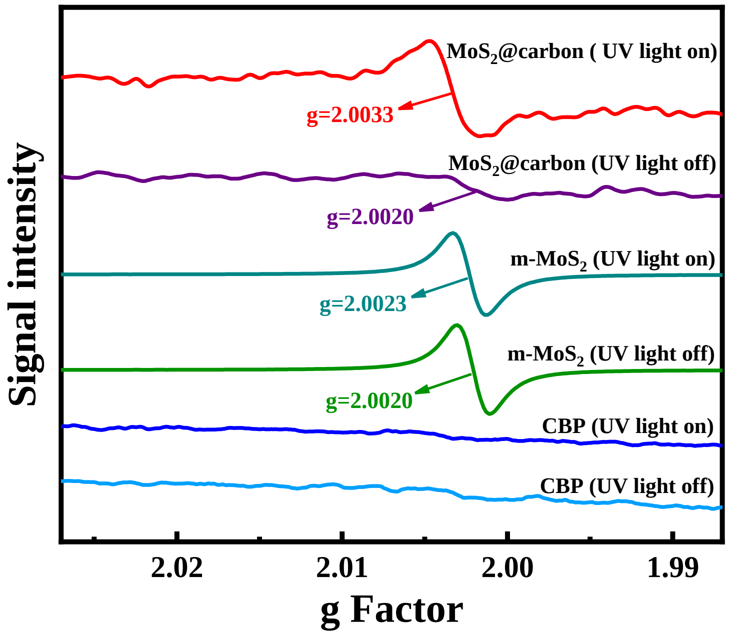

Figure S16. EPR curves of MoS_2@carbon, m-MoS_2, and CBP with and without 365 nm UV light illumination.

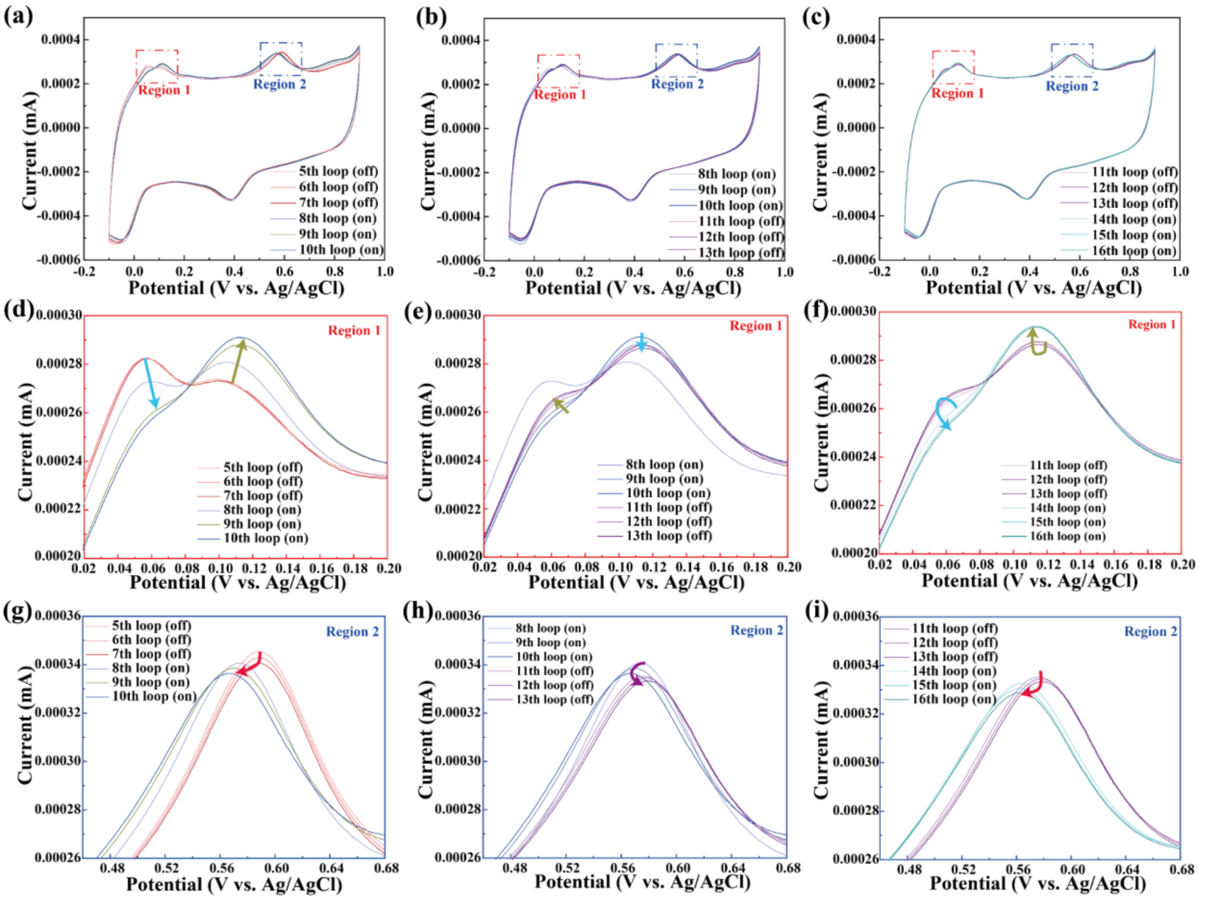

Figure S17. (a-c) CV curves of MoS_2@carbon tested during the switching of 365 nm UV light (5-16 loops, three-electrode system, scan rate: 5 mV s^{-1}), and (d-i) the magnified regions for the corresponding oxidation peaks.

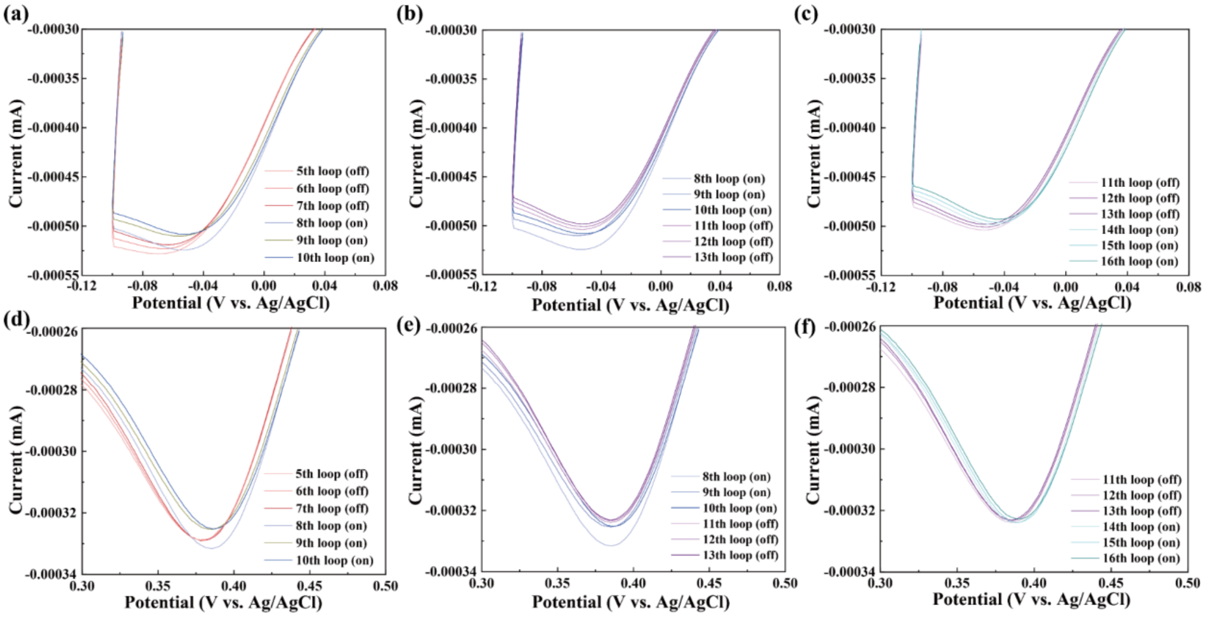

Figure S18. The reduction peak evolution of MoS_2@carbon tested in the three-electrode system during the switching of 365 nm UV light.

Table S1. Electrochemical performance of some recently reported MoS_2-carbon composites in the three-electrode system.

Materials	Electrolyte	Specific capacitance [F g^{-1}]	Current density [A g^{-1}]	Capacitance retention	Ref
MoS_2@Carbon	1 M H_2SO_4	1302	1	90.0% after 10000 cycles	This work
AFCNT-MoS_2	3 M KOH	516	0.5	82.4% after 5000 cycles	[7]
MoS_2@HCNB	6 M KOH	560	0.2	99.4% after 5000 cycles	[8]
1T-MoS_2 /carbon	1 M KOH	692	1	89% after 25000 cycles	[9]
MoS_2-CNT_{PPy}	1 M H_2SO_4	275	1	-	[10]
MoS_2/CNF	1 M KCl	903.9	1	94% after 5000 cycles	[11]
MoS_2@HCS-17	1 M Na_2SO_4	314.5	1	87% after 4000 cycles	[12]
MoS_2/MWCNT	0.5 M K_2SO_4	274.63	2	99.8% after 5000 cycles	[13]
MoS_2/N-CNTs-0.25	6 M KOH	220	1	89% after 10000 cycles	[14]
0.5-MoS_2@G /AC	3 M KOH	334	0.5	83.8% after 5000 cycles	[15]
CNT@MoS_2 /PDDA/PMo_{12}	1 M H_2SO_4	298	0.5	-	[16]

Table S2. Electrochemical performance of some recently reported asymmetric supercapacitors.

Asymmetric supercapacitors	Voltage window [V]	Energy density [Wh kg^{-1}]	Power density [W kg^{-1}]	Capacity retention (current density, cycles)	Ref
MoS_2@Carbon //AC	1.8	75.1	900	98.1% (10A g^{-1}, 10000)	This work
MoS_2-MoO_2/3DSG //FeS_2/3DSG	1.7	87.38	683.94	96.3% (8A g^{-1}, 10000)	[17]
p-MoS_2/NSG40 //NSG	1.8	65.2	900	91.7% (10A g^{-1}, 10000)	[18]
(Ni, Mo)S_2/G //NPG	1.6	84.5	500	91.8% (10A g^{-1}, 10000)	[19]
MoS_2/$NiCoS_4$@C //AC	1.6	53.01	4200	90.1% (10A g^{-1}, 10000)	[20]
MnO_2@OMCRs //OMCRs	2.0	55.2	200	75% (2 A g^{-1}, 5000)	[21]
NiHPi-500 //AC	1.5	50	362	100% (10 A g^{-1}, 5000)	[22]
NiFeP@$NiCo_2S_4$/CC //OPC-850	1.6	87.9	433.6	85.2% (8 A g^{-1}, 10000)	[23]
RuO_2 //Hex-Aza-COF-3	1.7	23.3	661.2	89% (5 A g^{-1}, 7500)	[24]
NiCoMn-S //AC	1.7	50.0	850.0	73.6% (10 A g^{-1}, 6000)	[25]

Table S3. Electrochemical performance of some recently reported symmetric supercapacitors.

Symmetric supercapacitors	Voltage window [V]	Energy density [Wh kg^{-1}]	Power density [W kg^{-1}]	Capacity retention (current density, cycles)	Ref
MoS_2@Carbon	1.4	91.7	700	89.9 % (10 A g^{-1}, 10000)	This work
TiC	1.6	41	2300	80.5 % (10 A g^{-1}, 20000)	[26]
MnO_2-Ag3/GO	2.0	57	1600	94 % (100 mV s^{-1}, 10000)	[27]
Mn_3O_4-WO_3	2.0	56.11	5000	95.5 % (10A g^{-1}, 5000)	[28]
NiS/MoS_2@N-rGO	0.4	35.69	601.8	94.5 % (10 A g^{-1}, 50000)	[29]
$CuCo_2O_4$-MnO_2	1.6	64.1	1500	80% (24 A g^{-1}, 5000)	[30]
graphene/MnV_2O_6	1	48.33	880.6	88 % (1 A g^{-1}, 3000)	[31]
ZnCo-LDH-2	1.5	40.3	15.08	93.3 % (1 A g^{-1}, 6000)	[32]
PCNs	3.0	65.8	300	92.8 % (1 A g^{-1}, 10000)	[33]
ZnO-Co_3O_4	1.6	39.3	19064.3	92.8 % (12.4 A g^{-1}, 10000)	[34]

References

[1] C. Yuan, T. Wu, J. Mao, T. Chen, Y. Li, M. Li, Y. Xu, B. Zeng, W. Luo, L. Yu, G. Zheng, L. Dai, *J. Am. Chem. Soc.* 2018, 140, 7629.

[2] S. Yang, S. Wang, X. Liu, L. Li, *Carbon* **2019**, *147*, 540.

[3] a) J. W. Kim, V. Augustyn, B. Dunn. *Adv. Energy Mater.* **2012**, *2*, 141; b) Z. Luo, L. Liu, X. Yang, X. Luo, P. Bi, Z. Fu, A. Pang, W. Li, Y. Yi, *ACS Appl. Mater. Interfaces* **2020**, *12*, 39098.

[4] a) Y. Gong, J. Wan, P. Zhou, X. Wang, J. Chen, K. Xu, *Mater. Sci. Semicond. Process.* **2021**, *123*, 105533; b) F. Qiao, W. Liu, S. Wang, F. Lin, Y. Chen, M. Yuan, Z. Weng, S. Wang, J. Zheng, Y. Zhao, *J. Alloys Compd.* **2021**, *870*, 159393.

[5] L. Xu, S. Xiong, S. Zhong, S. Bai, Y. Jiao, J. Chen, *Vacuum* **2020**, *174*, 109213.

[6] a) Y. Li, Z. Li, Y. Wu, H. Wu, H. Zhang, T. Wu, C. Yuan, Y. Xu, B. Zeng, L. Dai, *J. Power Sources* **2019**, *412*, 623; b) J. Dai, S. Zeng, Y. Lv, H. Xie, L. Luo, Y. Xu, L. Dai, *Electrochim. Acta* **2021**, *378*, 138149.

[7] H. Gupta, S. Mothkuri, R. McGlynn, D. Carolan, P. Maguire, D. Mariotti, P. K. Jain, T. N. Rao, G. Padmanabham, S. Chakrabarti, *Phys. Status Solidi A* **2020**, *217*, 1900855.

[8] L. Wang, F. Liu, B. Zhao, Y. Ning, L. Zhang, R. Bradley, W. Wu, *ACS Appl. Nano Mater.* **2020**, *3*, 6448.

[9] N. Joseph, C. B. A., *J. Electroanal. Chem.* **2020**, *874*, 114461.

[10] S. M. S. Pandiyarajan, G. K. Veerasubramani, R. M. Bhattarai, G. Gnanasekaran, S. J. Kim, Y. S. Mok. *ACS Appl. Energy Mater.* **2021**, *4*, 2218.

[11] A. Rajapriya, S. Keerthana, C. Viswanathan, N. Ponpandian, *J. Alloys Compd.* **2021**, *859*, 157771.

[12] K. Yang, A. Gao, H. Wu, F. Yi, D. Shu, X. Li, L. Xie, *Int. J. Energy Res.* **2020**, *44*, 7082.

[13] S. Radhakrishnan, S. R. K A, S. R. Kumar, P. Johari, C. S. Rout, *Nanotechnology* **2021**, *32*, 155403.

[14] W. Liu, W. Zhang, S. Zuo, C. Yao, X. Li, *J. Mater. Sci. Mater. Electron.* **2021**, *32*, 27184.

[15] H. Fu, X. Zhang, J. Fu, G. Shen, Y. Ding, Z. Chen, H. Du, *J. Alloys Compd.* **2020**, *829*, 154557.

[16] P. E. P. Win, J. Wang, X. Jia, B. Qi, W. Chen, L. He, Y. Song, *J. Alloys Compd.* **2020**, *844*, 156194.

[17] M. Zhang, Z. Song, H. Liu, A. Wang, S. Shao, *J. Colloid Interface Sci.* **2021**, *584*, 418.

[18] Z. Li, J. Zhao, Z. Yin, X. Wang, Z. Wu, D. Liu, M. Zhu, X. Wang, *Energy Fuels* **2021**, *35*, 2692.

[19] X. Yang, J. Mao, H. Niu, Q. Wang, K. Zhu, K. Ye, G. Wang, D. Cao, J. Yan, *Chem. Eng. J.* **2021**, *406*, 126713.

[20] Q. Li, W. Lu, Z. Li, J. Ning, Y. Zhong, Y. Hu, *Chem. Eng. J.* **2020**, *380*, 122544.

[21] P. Shang, J. Zhang, W. Tang, Q. Xu, S. Guo. *Adv. Funct.Mater.* **2016**, *26*, 7766.

[22] N. L. W. Septiani, Y. V. Kaneti, K. B. Fathoni, J. Wang, Y. Ide, B. Yuliarto, Nugraha, H. K. Dipojono, A. K. Nanjundan, D. Golberg, Y. Bando, Y. Yamauchi, *Nano Energy* **2020**, *67*, 104270.

[23] L. Wan, C. He, D. Chen, J. Liu, Y. Zhang, C. Du, M. Xie, J. Chen, *Chem. Eng. J.* **2020**, *399*, 125778.

[24] S. Kandambeth, J. Jia, H. Wu, V. S. Kale, P. T. Parvatkar, J. Czaban-Jóźwiak, S. Zhou, X. Xu, Z. O. Ameur, E. Abou-Hamad, A. Emwas, O. Shekhah, H. N. Alshareef, M. Eddaoudi, *Adv. Energy Mater.* **2020**, *10*, 2001673.

[25] C. Kang, L. Ma, Y. Chen, L. Fu, Q. Hu, C. Zhou, Q. Liu, *Chem. Eng. J.* **2022**, *427*, 131003.

[26] T. Chen, M. Li, S. Song, P. Kim, J. Bae, *Nano Energy* **2020**, *71*, 104549.

[27] V. J. Mane, S. B. Kale, S. B. Ubale, V. C. Lokhande, C. D. Lokhande, *Mater. Today Chem.* **2021**, *20*, 100473.

[28] S. Rudra, J. K, G Thamizharasan, M. Pradhan, B. Rani, N. K. Sahu, A. K. Nayak, *Electrochim. Acta* **2022**, *406*, 139870.

[29] X. Xu, W. Zhong, X. Zhang, J. Dou, Z. Xiong, Y. Sun, T. Wang, Y. Du, *J. Colloid Interface Sci.* **2019**, *543*, 147.

[30] K. Chanda, S. Maiti, S. Sarkar, P. Bairi, S. Thakur, K. Sardar, N. Besra, N. S. Das, K. K. Chattopadhyay, *ACS Appl. Nano Mater.* **2021**, *4*, 1420.

[31] W. H. Low, S. S. Lim, C. W. Siong, C. H. Chia, P. S. Khiew, *Ceram. Int.* **2021**, *47*, 9560.

[32] C. Wang, W. Wu, C. Zhao, T. Liu, L. Wang, J. Zhu, *J. Colloid Interface Sci.* **2021**, *602*, 177.

[33] Z. Song, D. Zhu, L. Li, T. Chen, H. Duan, Z. Wang, Y. Lv, W. Xiong, M. Liu, L. Gan, *J. Mater. Chem. A* **2019**, *7*, 1177.

[34] R. K. Mishra, G. J. Choi, H. J. Choi, J. Singh, F. S. Mirsafi, H. Rubahn, Y. K. Mishra, S. H. Lee, J. S. Gwag, *Chem. Eng. J.* **2022**, *427*, 131895.
